# Ultra-Processed Diets and Endocrine Disruption, Explanation of Missing Link in Rising Cancer Incidence Among Young Adults

**DOI:** 10.3390/cancers17132196

**Published:** 2025-06-29

**Authors:** Almir Fajkić, Orhan Lepara, Rijad Jahić, Almira Hadžović-Džuvo, Andrej Belančić, Alexander Chupin, Doris Pavković, Emina Karahmet Sher

**Affiliations:** 1 Department of Pathophysiology, Faculty of Medicine, University of Sarajevo, 71000 Sarajevo, Bosnia and Herzegovina; almir.fajkic@mf.unsa.ba; 2 Department of Human Physiology, Faculty of Medicine, University of Sarajevo, 71000 Sarajevo, Bosnia and Herzegovina; orhan.lepara@mf.unsa.ba (O.L.); almira.hadzovic@mf.unsa.ba (A.H.-D.); 3 University Clinical Center Sarajevo, 71000 Sarajevo, Bosnia and Herzegovina; rijadjahic2005@gmail.com; 4 Department of Basic and Clinical Pharmacology and Toxicology, Faculty of Medicine, University of Rijeka, Brace Branchetta 20, 51000 Rijeka, Croatia; andrej.belancic@uniri.hr; 5 Peoples’ Friendship University of Russia (RUDN University), Moscow 117198, Russia; chupin-al@rudn.ru; 6 International Society of Engineering Science and Technology, Nottingham NG11 8NE, UK; doris@viu.ba

**Keywords:** ultra-processed foods: endocrine-disrupting chemicals, early-onset cancer, microbiome, inflammation, epigenetics, Trojan horse model

## Abstract

The global increase in early-onset cancers among adolescents and young adults has happened at the same time as the rise in the consumption of ultra-processed foods (UPFs). Far beyond their poor nutritional quality, UPFs are increasingly seen as Trojan horses, complex biological agents that interfere with many functions of the human organism. In this review, we utilise the Trojan horse model to explain the quiet and building health risks from UPFs as foods that seem harmless, convenient, and affordable while secretly delivering endocrine-disrupting chemicals (EDCs), causing chronic low-grade inflammation, altering the microbiome, and producing epigenetic alterations. We bring together new proof showing that UPFs mess up hormonal signals, harm the body’s ability to fight off harmful germs, lead to an imbalance of microbes, and cause detrimental changes linked to cancer. Important components, such as bisphenols and phthalates, can migrate from containers into food, while additional ingredients and effects from cooking disrupt the normal balance of cells. These exposures are especially harmful during vulnerable developmental periods and may lay the groundwork for disease many years later. The Trojan horse model illustrates the hidden nature of UPF-related damage, not through a sudden toxin but via chronic dysregulation of metabolic, hormonal, and genetic control. This model changes focus from usual diet worries to a bigger-picture view of UPFs as causes of life-disrupting damage. Ultimately, this review aims to identify gaps in current knowledge and epidemiological approaches and highlight the need for multi-omics, long-term studies and personalised nutrition plans to assess and reduce the cancer risk associated with UPFs. Recognising UPFs as a silent disruptor is crucial in shaping public health policies and cancer prevention programs targeting younger people.

## Simple Summary

The alarming rise in early-onset cancers among adolescents and young adults parallels the global surge in ultra-processed food (UPF) consumption. Beyond poor nutrition, UPFs act as “Trojan horses,” introducing biologically active compounds, particularly endocrine-disrupting chemicals (EDCs), that interfere with hormonal regulation, immune responses, and microbial balance. These exposures, often occurring during vulnerable developmental stages, disrupt endocrine signalling; promote chronic, low-grade inflammation; alter the gut microbiota; and induce epigenetic changes, thereby creating a permissive environment for carcinogenesis. Key EDCs migrate from packaging into foods, while additives and high-temperature processing further compound the risk. This review integrates emerging evidence across disciplines to highlight UPFs as silent but systemic disruptors of metabolic and genetic homeostasis. The “Trojan horse” model reframes UPFs as long-term, multifactorial risk factors, underscoring the need for multi-omics research and personalised dietary strategies to assess and mitigate cancer risks in younger populations.

## 1. Introduction

In the 21st century, significant shifts in dietary patterns have occurred globally, with ultra-processed foods (UPFs) being a glaring example of this trend. UPFs belong to industrial formulations, mostly made up of quasi-main or minor substances extracted from foods (directly or indirectly), derived from food constituents, or synthesised in a laboratory. These have come to dominate the food landscapes of high- and middle-income countries [1]. Due to their convenience, affordability, longer shelf life, and aggressive marketing strategies, these products have become part of the daily diet of children, adolescents, and young adults (AYAs) worldwide.

The first issue that brought UPFs to public health attention was their malnutrition: they are characterised by excess amounts of added sugars, sodium, industrially produced trans fats, unhealthy fats, and a deficiency in dietary fibre, protein, and essential micronutrients. However, recent research over the last decade has transformed that knowledge, as the harmful health effects of UPFs extend beyond their macronutrient composition. These are chemically complex, biologically active systems whose interaction with the human body differs from minimally processed or whole foods [2].

Literature increasingly suggests that UPFs are liable to cause a broad range of non-communicable diseases. The significant points of difference between UPFs and other dietary risk factors are the hormonal homeostasis, immune regulation, microbial balance, oxidative stress, and gene expression they can affect [3]. Thus, UPFs are currently considered more as empty calories in disguise, but really are vehicles for biologically active compounds, particularly endocrine-disrupting chemicals (EDCs) such as phthalates, bisphenols, and nitrates, which mostly originate from food processing, packaging, or storage [4].

This review is primarily motivated by the troubling rise in the rates of early-onset cancers, that is, cancers diagnosed in AYAs. These include colorectal, breast, pancreatic, thyroid, and endometrial malignancies; elsewhere and across diverse population groups, their upward trends have been steadily documented. The conventional models of cancer risks based on ageing, genetic makeup, smoking status, and reproductive events may only partially explain such a magnitude of epidemiological transition. Therefore, scientists have turned their attention to new environmental and dietary exposures; UPFs come into the spotlight as one of the strong suspects.

The study’s premise suggests that UPFs may act as “Trojan horses” in oncogenesis; they might seem harmless due to palatability and convenience but harbour the potential to deliver carcinogenic compounds and create subtle yet persistent perturbations in human physiology. These effects could accumulate over time, particularly with exposure during critical periods of development, from infancy through adolescence into early adulthood, when endocrine, immune, and metabolic systems are still maturing.

UPFs are also believed to impact the gut microbiome, promoting dysbiosis, increased intestinal permeability, and systemic inflammation—factors increasingly implicated in carcinogenesis. Moreover, recent evidence points to their role in epigenetic reprogramming, whereby early or sustained exposure to food-based environmental cues can modify gene expression profiles without altering the DNA sequence, potentially predisposing individuals to malignancies years or even decades later [5].

Given the interdisciplinary nature of this emerging field, a comprehensive and integrative review is urgently needed. This paper aims to synthesise existing evidence on the role of UPFs in cancer development, and endocrine disruption will also be highlighted as a central mechanistic link.

## 2. Search Strategy

This narrative review was conducted to identify and analyse recent works related to the links between UPFs and cancer development in AYAs. Relevant articles published in the last 10 years were chosen using the PubMed and Scopus databases. Search terms included combinations of keywords using Boolean operators: (“ultra-processed food” OR “UPF”) AND (“cancer” OR “carcinogenesis” OR “neoplasms”) AND (“young adults” OR “adolescents” OR “early-onset cancer”) AND (“endocrine disruptors” OR “inflammation” OR “microbiome” OR “epigenetics”). Only full-text articles in English related to human studies were included. Studies were selected based on relevance to the mechanisms of endocrine disruption, metabolic dysfunction, and cancer pathways. Additional references were identified through manual screening of reference lists from key papers. The initial search yielded 312 publications. After removing duplicates and screening titles and abstracts for relevance, 127 studies were retained for full-text review. Of these, 64 studies were ultimately included in the synthesis based on their relevance to endocrine disruption, cancer development, and UPF consumption among adolescents and young adults.

## 3. Cancer in the Young Population

**Figure 1:**
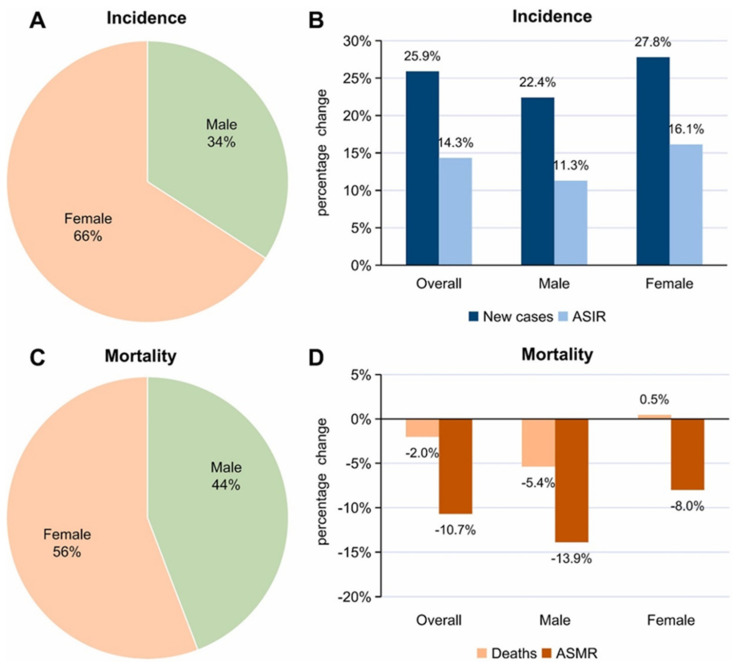
The estimated global new cases and cancer-related deaths in AYAs in 2022 compared to 2012. (**A**) Gender distribution of cancer incidence among Adolescents and Young Adults (AYAs) in 2022, (**B**) The chart shows the percentage change in new cancer cases and the Age-Standardized Incidence Rate (ASIR) from 2012 to 2022, (**C**) The chart shows the gender distribution of cancer-related deaths in AYAs in 2022, (**D**) The chart shows the percentage change in total cancer-related deaths and Age-Standardized Mortality Rate (ASMR) from 2012 to 2022. Legend: ASIR—age-standardised incidence rate; ASMR—age-standardised mortality rate. Reproduced with permission from Public Health; published by Elsevier. Copyright © 2023 Elsevier Ltd. All rights reserved [8].

The cancer epidemiology of young individuals, more specifically AYAs, differs in incidence, mortality, and types of cancer compared to other age groups. AYAs will be defined here as 15–39 years old; malignancies in this population are uniquely differentiated [6]. In 2022, around 1.3 million cases and 377.621 cancer deaths happened in AYAs; hence, the burden of cancer in this age group is significant (Figure 1). Incidence rates are more common in high-income countries, whereas mortality is higher in low-income countries, reflecting the inequalities in healthcare availability and outcomes. The cancers mostly seen in AYAs are breast cancer, cervical cancer, thyroid cancer, lymphomas, melanoma, and testicular and colorectal cancers. This is important since these are also the two main sites of cancer death for this age group [7].

Recent trends show that more AYAs are developing cancers just tied to obesity, like colorectal, breast, kidney, and pancreatic cancers. On the other hand, the rates of lung cancer (smoking-related cancer) and cervical cancer (infection-related cancer) have been going down. Obesity-related cancers are increasing in many countries, including the United States, the United Kingdom, Canada, Japan, South Korea, Australia, and the Netherlands [9].

Cancers in AYAs are associated with a complex interplay of lifestyle, socioeconomic factors, and biological factors. Smoking and alcohol consumption are key modifiable risk behaviours that continue to prevail in young males despite the decline in smoking trends. Lower socioeconomic status is connected to harmful behaviours of chronic diseases, such as inactivity and poor diet, which are associated with chronic inflammation. Early menarche and nulliparity reproductive factors expose this population more particularly to hormone-sensitive cancers such as breast cancer. Genetic predisposition plus exposure to carcinogens may increase risk on the one hand, and on the other, HPV-related cancers persist due to inadequate vaccination coverage [10]. UPFs promote obesity and cancer risk not only due to excess calories but also through endocrine-disrupting chemicals and additives that drive inflammation, metabolic dysfunction, and microbiome changes. However, recent large-scale randomised trials of metformin, a diabetes drug targeting metabolic pathways implicated in obesity-related cancers, have shown no significant reduction in cancer incidence or improved outcomes in most cancer types, highlighting the complexity of metabolic interventions and the need for more targeted mechanistic research in UPF-associated carcinogenesis [11].

In addition, obesity is increasingly being seen as a very alarming trend in this population, previously reported to be associated with thyroid, breast, and endometrial cancers [12]. In addition to the standard dietary explanations, the impact of UPFs, industrial preparations that have become a significant part of modern diets, is gaining considerable attention. Such foods are high in energy value but low in nutrition and contain many additives that have been linked to inflammation, endocrine disruption, and metabolic diseases. Hence, UPFs might be an implicit major contributor to the factors associated with obesity and cancer in the AYA population. Teenagers and AYAs often obtain a significant portion of their daily calories from UPFs. In some studies, it was noted that over half of the energy consumed by adolescents in the US and UK comes from this food type (Table 1). The significant variation (25–67.8% UPF energy intake) reflects the different eating patterns of a country, the extent to which the food industry has influenced them, differences in regulations, and cultural preferences. For instance, the lower UPF contribution in Kenya (~25%) likely reflects a greater reliance on traditional, minimally processed staple foods and limited penetration of the packaged food industry. In contrast, studies from Brazil, the UK, and the US reported substantially higher UPF intake, with the UK reaching 67.8% among adolescents. This disparity likely stems from increased urbanisation, greater availability and marketing of ultra-processed products, and dietary shifts away from home-cooked meals in higher-income or rapidly industrialising settings [13,14,15,16,17,18,19,20,21,22]. This fact highlights the role of UPFs in metabolic risk early in life.

The latest data from the extensive prospective UK Biobank study further confirms the existence of a systemic yet concealed threat associated with UPF consumption. An increase of just 10% in the proportion of UPFs in the overall diet was statistically significantly associated with a higher incidence and mortality from several types of cancer, including ovarian cancer, brain cancer, and lymphomas, even after adjustment for traditional risk factors [13,23].

## 4. UPFs Are Beyond Poor Nutrition

A UPF is a commercial product that contains little to no intact foods and is composed of modified and unmodified ingredients taken out of food. These foods are far more than just junk food; they include a wide variety of foods. Junk food has never been defined scientifically or specifically; consumers usually refer to manufactured confections, fast food, and salty, fatty, and/or sugary snacks, but the description is still ambiguous. Furthermore, UPFs encompass a range of options, including industrial organic, plant-based (such as those found in a vegan diet), gluten-free, micronutrient-enriched, and low-calorie foods. These foods are frequently marketed as healthy to consumers and are commonly rated as such by compositional food indices, such as the Australian Health Star Rating Labelling System, the French Nutri-Score, and the English Traffic Light System [24,25]. These front-of-pack ratings, though, are solely derived from nutrient density and not the processing intensity, which might lead to misclassification of industrially formulated foods [26,27,28].

While UPFs are often considered homogeneously unhealthy, they comprise a broad spectrum of products and include plant-based analogues. These are industrially formulated meat, dairy, and egg substitutes composed mainly of components derived from plants, including protein isolates, refined oils, and starches, often with added texturisers, flavourings, and preservatives to approximate animal-based counterparts [29]. Although they belong to UPFs due to their intensive processing, the nutritional quality is what greatly separates them from typical UPFs, such as sugar-sweetened beverages, packaged snacks, processed meats, and ready-to-eat meals [30]. Both plant-based and conventional UPFs are typically energy-dense, with added sugars, salt, and fats, though the exact nutrient profiles vary [31]. Most importantly, recent cohort studies have indicated that both plant- and animal-based UPFs are associated with higher risks of all-cause and cancer mortality, underscoring the imperative need for health impact evaluation beyond source ingredients alone [3].

UPFs typically include a wide range of food additives—such as artificial sweeteners, colourants, preservatives, and emulsifiers—that enhance palatability and shelf life. Notably, emulsifiers such as carboxymethylcellulose and polysorbate-80 have been implicated in disrupting gut barrier function and promoting intestinal inflammation and tumorigenesis in animal models, particularly in the context of colorectal cancer [32]. More than half of the total energy value consumed in high-income nations (the US and UK) and between one-fifth and one-third of the total energy value consumed in middle-income countries already come from ultra-processed foods. Sales of these products often increase by 1% annually in high-income nations and by up to 10% in middle-income nations [26,33].

In a scientific context, ultra-processed foods were first introduced as part of the NOVA classification system. NOVA categorises foods into four mutually exclusive classes based on the extent and purpose of their industrial processing: (1) “unprocessed or minimally processed foods,” encompassing fresh, dried, or frozen fruits and vegetables, cereals, legumes, meat, fish, and dairy; (2) “processed culinary materials,” which encompass table sugar, oils, fats, salt, and other substances derived from food or nature, utilised in kitchens for culinary preparations; (3) “processed foods,” encompassing items such as canned fish, vegetables, basic bread, and cheeses, created by incorporating salt, sugar, oil, or other culinary additives into uncooked or minimally processed meals; and (4) “ultra-processed foods,” which are concoctions of ingredients derived from various industrial processes and mostly solely used in industry [34,35].

Despite the lack of a widely accepted definition, food processing can be broadly defined as any deliberate alteration made to food between its place of origin (i.e., the manufacture of raw foods or food ingredients) and its point of destination (i.e., the consumption of a completed food product). Therefore, the scope and goal of modern food processing can vary. Washing, grinding, mixing, chilling, storing, heating, freezing, filtering, fermenting, extracting, extruding, centrifuging, frying, drying, hydrogenating, concentrating, pressurising, irradiating, microwaving, and packaging raw food, for instance, can be as easy as that [2].

Food processing, which includes a wide range of physical and chemical changes, is essential in food preparation. Temperature, pressure, and the presence of reactive agents are the primary drivers of the physical and chemical changes during processing [36]. Without a doubt, processing affects the nutrient content and bioavailability of food, which in turn significantly influences how consumers perceive a food product’s healthfulness. The perceived health benefits of raw food are reduced, and its perceived calorie content is increased by even basic mechanical processing [2]. While evidence linking UPFs to early-onset cancer is growing, most supporting data come from observational studies subject to confounding and cannot establish causality. Additionally, reliance on self-reported dietary intake may introduce recall bias and misclassification, limiting the precision of current exposure assessments. Therefore, high-quality, longitudinal studies are needed to draw more definitive conclusions across all mechanistic and epidemiological aspects.

Food processing can significantly affect its nutritional quality, either positively or negatively. Key ways in which this occurs include nutrient loss; physical removal of vitamins, minerals, and fibre; chemical degradation such as the oxidation of vitamins and carotenoids during fruit and vegetable processing; loss of minerals; and changes in the bioavailability of nutrients. Processing can increase or decrease the body’s ability to absorb nutrients (higher bioavailability of lycopene in tomato pastes); form harmful compounds, such as acrylamide and polycyclic aromatic hydrocarbons, during high-temperature processing; reduce anti-nutritional factors, such as phytic acid, lectins, and cyanogenic glycosides, during cooking and dipping; or consist of fortification, the addition of essential micronutrients lost during processing (enrichment of flour with folic acid and iron), or chemical transformation, the production of functional ingredients (glucose and fructose syrups, emulsifiers) [37].

On the other hand, eating ultra-processed products (UPPs) lowers intake of micronutrients and dietary diversity. According to specific epidemiological research, diets high in UPPs are not particularly nutritious. Energy density, added sugar, total fat, and saturated fat all rise with consumption of UPPs, while the content of proteins, dietary fibre, dietary diversity, micronutrients (like niacin, folate, vitamin B12), trace elements (zinc), and essential minerals (calcium, magnesium, potassium, phosphorus) falls [26].

A continuous scientific discussion exists regarding the relationship between food processing levels and traditional nutrient-focused metrics and health. Some academics also argue that ultra-processed meals are unhealthy simply because they are low in nutrients, casting doubt on the value of focusing on the processing level beyond traditional nutrient-centric classification schemes. Nutrient profile indices, such as the Healthy Eating Index, the Nutri-Score, and the Nutrient-Rich Food Index, reveal an inverse relationship between UPF intake and diet quality [35].

In a large sample of Italians, a diet high in UPFs was linked to an acceleration of biological ageing. This link was not well explained by the low nutritional makeup of highly processed foods, indicating that the non-nutrient qualities of these foods may have a negative impact on biological ageing [38]. High intake of UPFs is associated with an increased risk of illnesses, diseases, and early death, according to research from numerous nations. Obesity and adiposity outcomes have been associated with cardiovascular disease (CVD); breast, ovarian, brain, and overall cancer; non-alcoholic cirrhosis; Crohn’s disease; chronic renal failure; depression; cognitive decline and dementia; type 2 diabetes; hypertension; dyslipidaemia; hyperuricemia; and all-cause mortality [30].

In recent times, an increasing number of researchers have focused on examining the influence of UPFs on the endocrine system, as they represent a potential source of EDCs [39,40]. In intact organisms, their offspring, or (sub)populations, endocrine disruptors are defined as “exogenous substances or mixtures that alter the functions of the endocrine system and consequently cause adverse health effects” [41]. The neuroendocrine system, thyroid function, prostate and breast cancer risk, and the male and female reproductive systems are all adversely affected by exogenous substances that can imitate, block, or interfere with endocrine hormones. Because these chemicals can enter the food supply through environmental sources, including animal feed, fertilisers, contaminated soil and groundwater, and food contact items such as plastic tubing, conveyor belts, and packaging materials, diet is a significant source of EDC exposure [39].

## 5. Endocrine Disruptors in UPFs and Their Systemic Impact

To describe the mechanistic link between UPFs and cancer onset, exploring their role as carriers of endocrine-disrupting chemicals (EDCs) is crucial. UPFs provide a primary dietary source of EDCs, which are exogenous compounds that can interfere with hormone signalling. Packaging these foods adds contamination (bisphenols and phthalates), additives, and raw ingredients that may be tainted. As actual mimics or blockers of hormones, most commonly multiple sites of receptor binding, gene transcription, and even epigenetic regulation become hormonally dysregulated. More evidence continues to show that exposure to EDCs via UPFs may lead to hormonal perturbations in estrogenic, androgenic, and glucocorticoid pathways, with the outcome of metabolic diseases plus hormone-related cancers. EDCs often exhibit non-monotonic dose–response relationships, so low dietary exposures through UPFs may have disproportionately significant biological effects, especially if they occur during hormonal-sensitive developmental stages, such as adolescence [42].

EDCs comprise artificial and naturally occurring chemicals in the environment (air, water, and soil) and in medications, food supplies, cosmetics, and manufactured products [43]. In defining EDCs, their primary interaction with the endocrine system must be considered. In this system, hormone receptors located either on the cell membrane, in the cytoplasm, or in the nucleus act as membrane transporters, assisting the entry of hormones into target cells. They may also be present as serum proteins that distribute hormones and catalyse enzymes related to hormone synthesis and inactivation. This interference can result in numerous symptoms and manifestations [44].

Recently, several countries have strived to control EDCs by legislating a ban on the major compounds. For example, bisphenol A (BPA) was banned from the European Union market for plastics related to food, e.g., baby bottles. This has since extended to plastic receipts from cash registers [45]. It is nearly impossible to know all the molecules unrecognised as endocrine disruptors; for instance, about 4000 ingredients in tobacco products are allowed in the EU, despite evidence that they contain EDCs, one of which is cadmium. As a heavy metal, cadmium is highly atherogenic, and it contributes to plaque formation in arteries. Its effects may last for many years and possibly for generations to come. Hence, the outcomes of cadmium exposure may be direct, like in carcinogenic pathways, or indirect, like in obesogens, leading eventually to chronic conditions such as hypertension and type 2 diabetes mellitus [46].

An exciting feature is the ability of some metabolic wastes in the human body, such as cortisol, to act as EDCs by increased in-house production or external administration of related compounds. These molecules attach to specific sites, facilitating the release of more hormones [39]. Problems with how they stick can lead to homeostatic imbalances, causing faulty hormone output, such as in metabolic syndrome, or because antibodies destroy the receptors, as seen in Graves’ disease [47]. To fully understand EDCs, all interactions must be considered because, like non-endocrine organs, the heart also produces several different hormones. For example, the heart releases atrial natriuretic peptide and its subtypes—brain natriuretic peptide and C-type natriuretic peptide [48].

**Figure 2:**
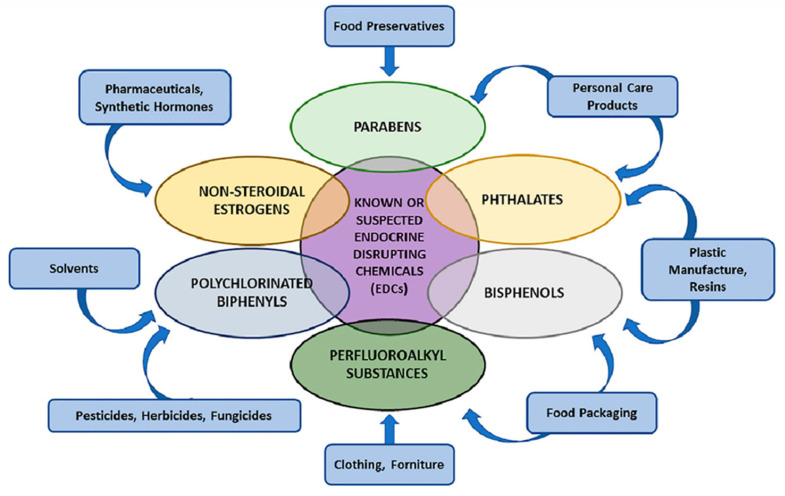
EDCs’ chemical characteristics and classification. Reproduced with permission from Int. J. Mol. Sci.; published by MDPI, 2020 [51].

The classification and categorisation of EDCs remains an open, debatable issue. One pattern of classification based on causative effects is carcinogens, obesogens, disruptors, and stressogens [49]. Another pattern of classification established in the sources of EDCs is pharmaceuticals, personal care products, industrial chemicals, and agricultural chemicals. Such classifications tend to overlook essential EDCs, such as metals like cadmium, lead, mercury, and arsenic. The specific category called “inner body EDCs” includes hormones and peptides naturally synthesised within the human body (progesterone, testosterone, cortisol, and estrone) [50]. A proposed regulated classification for EDCs in specific contexts is provided in Figure 2.

The human metabolic system uses enzymes to convert EDCs. The cytochrome P450 family of enzymes mostly plays an essential role in metabolising many foreign chemicals and body compounds. Most importantly, there should be concern about this enzyme family’s interaction with the metabolism of potent medicines, such as chemotherapy drugs [52]. It is also likely that EDCs exhibit different responses between men and women due to differences in receptor expression, such as oestrogen receptors and their effects on breast health and cancer formation, as well as variations in cytochrome P450 family genes and their methylation capabilities [53].

The aryl hydrocarbon receptor (AHR), a transcriptional control protein encoded by the AHR gene located on chromosome 7, is influenced by both internal and external binding molecules, including bilirubin, prostaglandin G2, tryptamine, omeprazole, and mitogen-activated protein kinase 1, as it is present in most cells [54]. Therefore, it has crucial functions in the response to injury removal and immune response actions. In 1969, Poland et al. were the first to show that 2,3,7,8-tetrachlorodibenzo-p-dioxin, identified as an EDC, could act as a binding molecule and activator of AHR. The AHR forms a complex with member CYP1 of the cytochrome P450 family enzyme CYP450Ia, responsible mainly for the catalysis of mutagenic compounds and deactivation of numerous anticancer drugs. This family may also be affected by estradiol, adding to the effects of EDCs, with cytochrome P4501A1 being mainly an extrahepatic monooxygenase located in the lungs, kidneys, and skin [55]. The most obvious and direct ways EDCs affect UPFs through the AHR are by di-isononyl phthalate (DINP), di(2-ethylhexyl)phthalate (DEHP), cadmium (Cd), lead (Pb), arsenic (As), and mercury (Hg.) These chemicals do not just initiate cancers; in multiple sclerosis models, it has also been proven that EDCs trigger the IRE1 alpha-XBP1 signalling pathway [56]. Certain gene variations, such as the Phe318 mutation, can result in a total loss of AHR activity, whereas other mutations do not significantly impair function. The AHR is found mainly in the cytoplasm in a form that binds ligands associated with heat shock protein 90 and immunophilin-like X-associated protein 2 [57]. For a while, many researchers attempted to define the exact cause that led to the dissociation of this complex and the entry of the AHR into a nucleus. In particular, investigating the complex, such as AHR and the heat shock protein 90 inhibitors, could be a key for inactivating the AHR in terms of exogenous ligands using 17-allylaminodemethoxygeldanamycin and celastrol [58].

Phthalates, as plasticisers, are synthetic chemical compounds added to polyvinyl chloride (PVC) to make it a flexible, strong, and clear form. The most significant concern is the presence of phthalates in food contact materials, such as packaging, processing tools, and containers, as they have been found to have alarmingly high levels in highly processed foods. Practical, immobile forms (such as refined compounds, additives, and preservatives) create easy pathways for phthalate contamination. DEHP and DINP are typically detected in UPFs. Phthalates are susceptible to translocation into the bloodstream because they are not covalently bonded to the polymer matrix in plastics exposed to high temperatures, extended storage periods, and high fat content, all of which are frequently present in UPFs, speeding up this migration. Due to their characteristic lipophilicity, phthalates concentrate in fat-containing materials, making UPFs high in fat (such as processed meats, dairy desserts, snack foods, and baked goods) more likely to contain phthalates in higher concentrations than their counterparts that are less processed [59,60].

Studies have shown that high intake of UPFs is associated with high urinary levels of metabolites of phthalates and bisphenols, which implies higher exposure to these EDCs. For instance, Baker et al. found that a greater dietary proportion of UPFs was associated with higher concentrations of di(2-ethylhexyl) phthalate metabolites among pregnant women and that these foods may contribute to socioeconomic disparities in phthalate exposure [60]. Martínez Steele et al. found that urine levels of different phthalates and bisphenols increased with more intake of UPFs in a nationally typical group of the US population [15].

When UPFs are manufactured, packaged, and stored, they are often exposed to BPA, one of the most common endocrine-disrupting chemicals (EDCs). Migration from plastic containers is mainly accomplished by heating (e.g., leaching from epoxy resin linings in canned foodstuffs, especially acidic, salty, and fatty products, or heating in a microwave), and excessive consumption of ultra-processed foods has been linked to elevated BPA levels in urine, according to research where it was found that BPA was noticed to be higher in UPF users [61].

BPA and its analogues are known to interfere with endocrine function by mimicking oestrogen and binding to oestrogen receptors, which can disrupt hormonal balance and contribute to obesity and cancer risk. BPA has been shown to promote adipogenesis and increase the expression of proinflammatory cytokines such as IL-6, IL-1β, and TNF-α, which are associated with a chronic low-grade inflammatory state typical of obesity. This inflammation can create a cancer-promoting environment, particularly in estrogen-responsive tissues like the breast and colorectal tissues [62,63].

In breast cancer, BPA exposure has been linked to the progression of cancer through mechanisms such as the CXCL12/AKT signalling pathway, which promotes epithelial–mesenchymal transition (EMT) and cancer cell migration. Similarly, in colorectal cancer, BPA exacerbates cancer progression in the context of obesity by activating pathways like PI3K-AKT, which are involved in inflammation and receptor activation [64]. Furthermore, BPA and its analogues can disrupt insulin signalling and fatty acid metabolism, contributing to insulin resistance and metabolic dysfunction, which are risk factors for both obesity and cancer. The presence of these EDCs in UPFs underscores the potential health risks associated with their consumption, particularly in vulnerable populations such as those with obesity [65].

BPA, a known modulator of the oestrogen receptor, may interact with the AhR directly or indirectly through other signalling pathways, according to specific in vitro and in vivo investigations. These studies found that BPA increased AhR-dependent gene expression by binding to AhR less firmly than conventional EDC ligands. BPA may indirectly modulate AhR activity by interfering with the ER-AhR crosstalk, changing the redox state of cells, and influencing co-regulatory proteins shared by the ER and AhR pathways, while developmental toxicity, immunological modulation, altered gene expression, and synergistic toxicity are some of the downstream effects of the interaction between BPA and AhR. Interference between BPA and the AhR during critical times may impair normal development, and coactivation or interference between BPA and other AhR ligands may intensify toxic effects [66,67]. According to supporting data, BPA partially induces CYP1A1 mRNA in liver and breast cancer cell lines through the AhR pathway, increasing the nuclear translocation of AhR and enhancing CYP1A1 expression in the liver and gut. Also, BPA levels correlate with immune and detoxification gene expression patterns consistent with AHR modulation [68,69].

The global sourcing of ingredients further complicates regulation and traceability for phthalate contamination. UPFs are processed foods often made by mixing different ingredients and heavy additives. These additives reveal much about Cd, Pb, As, and Hg, particularly in the case of cocoa powder, as this is a common ingredient in ultra-processed snacks and drinks. These snacks must suddenly join multiple points of the food chain, hence posing a danger to public health [70,71].

The processing of these foods can lead to the formation of advanced glycation end products (AGEs). As proposed endocrine disruptors, they induce oxidative stress and disturb the hormonal balance. This, therefore, underscores the need for policies to minimise dietary exposure to synthetic chemicals through improved food packaging and processing practices [72]. AGEs are formed through non-enzymatic glycation reactions, primarily via the Maillard reaction, which occurs during the cooking and processing of foods at high temperatures. These compounds have been implicated in various chronic diseases, such as diabetes, cardiovascular diseases, and neurodegenerative disorders [73]. Moreover, the bioavailability and metabolic fate of AGEs depend on their form, whether free or bound. Free AGEs exhibit greater absorption efficacy and can impact cellular energy homeostasis by disrupting mitochondrial function, as well as pathways of energy production, including the AMPK-SIRT6 signalling pathway. This may lead to mitochondrial dysfunction, aggravating metabolic and endocrine disorders [74].

Together, the literature data show that UPFs are a significant and changing source of EDCs. By messing with basic hormone pathways, these exposures may sneakily help the growing number of hormone-related illnesses. This hormonal disruption accounts for just a small part of the greater physiological effects of UPFs. More and more evidence shows that UPFs may cause even more serious molecular damage, especially to the gut microbiome and epigenome, in ways that can quietly rewire body systems and lead to increased long-term cancer risk.

## 6. Mechanistic Pathways Linking Ultra-Processed Foods to Cancer

### 6.1. UPF-Induced Microbiome Disruption and Its Implications

UPFs are industrial formulations with minimal whole-food content and often contain additives that significantly impact gut microbiota composition, potentially leading to microbial dysbiosis—a condition increasingly implicated in carcinogenesis [75]. Understanding how UPFs modulate the gut microbiome (GM) and how dysbiosis may link to cancer development is essential for developing practical nutritional guidelines and preventive strategies.

The human GM comprises trillions of microorganisms that contribute to numerous physiological functions, including nutrient metabolism, immune modulation, and maintenance of epithelial integrity. Short-chain fatty acids (SCFAs), such as butyrate, acetate, and propionate, are produced by microbial fermentation of dietary fibre and play pivotal roles in anti-inflammatory processes and colonocyte energy metabolism [76]. A well-balanced GM supports the gut barrier, enhances mucosal immunity, and helps in preventing colonisation by pathogenic bacteria. Alterations in gut microbiome (GM) diversity and composition—commonly termed dysbiosis—are associated with systemic inflammation, metabolic dysfunction, and increased cancer risk [77].

**Figure 3:**
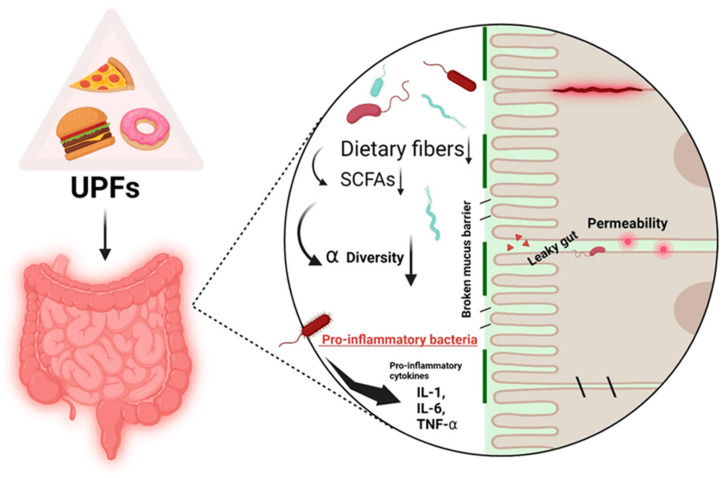
Mechanisms linking ultra-processed foods to microbiome dysbiosis. Reproduced with permission from Nutrients; published by MDPI, 2025 [5].

UPFs typically lack fermentable fibres and are rich in components that can affect GM composition (Figure 3). Emulsifiers, such as polysorbate-80 and carboxymethylcellulose, have been shown to degrade the mucus layer and increase intestinal permeability in animal models [78]. Additionally, artificial sweeteners such as saccharin and sucralose have been reported to shift microbial profiles towards proinflammatory species. Individuals with high UPF intake exhibit significant reductions in beneficial SCFA-producing taxa such as *Faecalibacterium prausnitzii*, *Roseburia hominis*, and *Butyribacter* sp. These individuals also display enrichment in Blautia_A and Clostridium symbiosum—microbes implicated in proinflammatory states and cancer metabolism [77,79].

Besides these main mechanisms, the “endobolome” concept has been introduced to describe the group of GM genes and pathways involved in the metabolism of steroid hormones and EDCs. Dysbiosis and reduced microbial variety may alter endobolome functions, contributing to hormonal imbalance and the development of pathophysiological states. This adds even more complexity to how UPFs change the microbiome, altering cancer risk and highlighting another potential way for microbiome-targeted interventions to mitigate these effects [80].

Dysbiosis contributes to carcinogenesis through various interconnected mechanisms, especially in colorectal cancer (CRC), which represents one of the most strongly microbiome-associated malignancies. Among the primary consequences of UPF-induced dysbiosis is a marked reduction in the production of short-chain fatty acids (SCFAs), particularly butyrate, due to the loss of fibre-fermenting commensals, such as *Faecalibacterium prausnitzii* and *Roseburia* spp. Butyrate is a critical energy source for colonocytes, enhances epithelial integrity, and functions as a histone deacetylase (HDAC) inhibitor, modulating gene expression and promoting apoptosis of transformed cells [81]. A deficiency in butyrate leads to epigenetic instability, barrier dysfunction, and enhanced proinflammatory signalling, creating a tumour-permissive microenvironment. Although propionate also has anti-inflammatory roles, its reduction in UPF-dominated diets contributes to compromised immune regulation and mucosal vulnerability [77].

UPF-associated dysbiosis elevates the generation of pro-carcinogenic metabolites (Table 2). For instance, hydrogen sulphide (H*_2_*S), produced by sulphate-reducing bacteria such as Desulfovibrio spp., has been shown to exert genotoxic effects through mitochondrial damage, oxidative stress, and impaired stem cell renewal in the gut epithelium [82]. Furthermore, dysbiosis drives the conversion of primary into secondary bile acids, including deoxycholic acid (DCA) and lithocholic acid (LCA), which can induce DNA damage and oxidative stress and activate proliferative pathways such as the Wnt/β-catenin and EGFR signalling pathways [83].

A study by Yang et al. demonstrated that trimethylamine N-oxide (TMAO), a gut microbiota-derived metabolite produced from dietary precursors such as choline and carnitine, exerts oncogenic effects by promoting CRC cell proliferation and angiogenesis, thereby contributing to disease progression [84]. Additionally, lipopolysaccharide (LPS), a structural component of Gram-negative bacterial membranes, is present in higher levels in UPF-induced dysbiosis. LPS activates Toll-like receptor 4 (TLR4), leading to NF-κB activation and the subsequent transcription of inflammatory mediators such as IL-6 and TNF-α, thereby fostering chronic low-grade inflammation and tumour progression [85]. These microbial shifts and metabolic imbalances form a bridge between ultra-processed food consumption and colorectal carcinogenesis, supporting the concept of diet-induced microbiome-mediated cancer risk.

UPFs negatively alter the gut microbiome and create a metabolic environment that can contribute to carcinogenesis. These findings stress the importance of dietary interventions to minimise UPF consumption and restore microbial balance. Future studies should be aimed at identifying early biomarkers of dysbiosis-induced cancer risk. Strategies such as prebiotic/probiotic supplementation and personalised dietary regimens may hold therapeutic promise [77,86].

In vitro models, including batch fermentations and colonic simulators inoculated with human faecal microbiota, show that UPF ingredients—particularly emulsifiers like carboxymethylcellulose or, more broadly, carboxymethylcellulose-based emulsifiers along with polysorbate-80, artificial sweeteners like sucralose or acesulfame potassium, preservatives such as potassium sorbate and sodium benzoate, and nanoparticles like titanium dioxide—greatly worsen microbial diversity and the structure of communities [87,88]. These compounds alter microbial community composition to increase harmful bacteria while decreasing beneficial health-related bacteria such as *Faecalibacterium prausnitzii* and promoting the growth of Roseburria in greater detail. Additionally, they enhance *Enterococcus* and *Veillonella* species that can promote inflammation. Related shifts include decreased SCFA synthesis, a lowered barrier function of the mucosa, and increased pro-inflammatory metabolite generation [89].

Mechanistic in vitro data also indicate that the introduction of these additives impairs mucin expression, increases markers of intestinal permeability, and activates microbial pathways associated with low inflammation [5]. While these findings have been made in controlled systems, they point consistently to the fact that components of UPFs lead to dysbiosis and disrupt the balance of microbes.

In vivo studies in both animal and human trials confirm the findings and provide additional information by considering host responses. Rodent models given a diet rich in UPFs or with additives show low microbial diversity, high pro-inflammatory markers such as TNF-α and IL-6, low SCFA levels, and poor gut barrier condition [90]. For instance, in a controlled human intervention study, results showed that 11 days of consumption of carboxymethylcellulose caused significant reductions in the richness of microbes as well as reduced faecal SCFAs, and for some participants, it also triggered bacterial infiltration into the mucus layer, which is an indication of barrier dysfunction [91].

A significant contrast between in vitro and in vivo studies is their time frames and biological complexity. In vitro experiments typically last for hours or days at most; while very useful for mechanistic insights, they do not develop microbe–host interactions nor provide metabolic contexts [92,93]. In vivo interventions last from several days to a few weeks (2–4 weeks in human trials) to more accurately reproduce real-world exposure and adaptation processes [94,95]. Still, outcome results could be modulated by inter-individual variation, dietary background, and microbiome resilience when determined in vivo [96].

The convergent in vitro and in vivo evidence supports the conclusion that UPFs, through both their nutrient composition and additives, have a negative impact on the gut microbiome. Such perturbed states of low diversity, dysbiosis, and the resultant loss of beneficial microbial metabolites may, in turn, promote systemic inflammation, insulin resistance, and a host of chronic diseases [5]. Further translational studies should develop an approach to determine the long-term effects of UPF components, as well as their interactions with host genetics and microbiota, and also investigate reversible changes induced by dietary modulation.

### 6.2. Epigenetic Reprogramming, Silent Link Between UPFs and Cancer

Epigenetic modifications, heritable yet reversible alterations in gene expression that do not involve changes to the underlying DNA sequence, are increasingly recognised as pivotal regulators in cancer development. These modifications include DNA methylation, histone tail modifications, chromatin remodelling, nucleosome positioning, and post-transcriptional regulation by non-coding RNAs, particularly microRNAs. Collectively, these mechanisms orchestrate gene regulatory networks that govern cell fate, differentiation, and genome stability. In the context of cancer, aberrant epigenetic reprogramming can lead to the silencing of tumour suppressor genes or the activation of oncogenic signalling pathways, often occurring early in tumorigenesis. These changes do not require mutational damage but instead act by reconfiguring the transcriptional landscape of cells, thereby driving malignant transformation. Persistent patterns of global hypomethylation and promoter-specific hypermethylation have been implicated across diverse malignancies and are increasingly proposed as biomarkers for early detection, risk stratification, and therapeutic targeting [97].

The expanding field of nutriepigenomics has illuminated the profound impact of diet on the epigenome. Several bioactive dietary compounds have demonstrated the ability to influence chromatin architecture and gene expression by modulating the activity of epigenetic enzymes, including histone deacetylases (HDACs), histone acetyltransferases (HATs), DNA methyltransferases (DNMTs), and regulators of chromatin remodelling and post-translational histone modifications. These interactions are not merely transient; they shape cellular memory and may exert long-term effects on cancer susceptibility, particularly during critical developmental periods [98].

Mounting evidence suggests that UPFs are not only calorically dense and nutritionally imbalanced but also serve as vehicles for epigenetically active compounds capable of disrupting gene regulation. Thus, it is unsurprising that UPF-derived compounds have been shown to influence DNA methylation patterns, histone modification profiles, and microRNA expression in ways that mimic or exacerbate known oncogenic epigenetic signatures. Chronic exposure to UPFs, particularly during critical developmental times, may induce lasting epigenetic reprogramming that increases cancer susceptibility—a mechanistic insight that aligns with and potentially explains the rising incidence of malignancies among progressively younger populations [99,100]. To fully elucidate the carcinogenic potential of ultra-processed diets, it is essential to dissect the underlying epigenetic mechanisms through which nutritional exposures modulate gene expression and cellular behaviour [101].

Among the various epigenetic modifications, DNA methylation remains the most extensively studied, not necessarily due to its prevalence but because of the relative ease with which it can be analysed. This process entails the covalent addition of a methyl group (CH_3_) to the 5-position of cytosine residues within CpG dinucleotides—sites that, despite comprising only ~1% of the human genome, account for over one-third of all point mutations implicated in genetic disorders. CpG methylation occurs across intergenic regions and repetitive elements and in CpG-dense promoter regions of genes regulating critical cellular functions such as proliferation, differentiation, and DNA repair. When occurring within gene promoters, hypermethylation is frequently associated with transcriptional silencing, particularly of tumour suppressor genes and DNA repair pathways, and is a well-established hallmark of many cancers [102].

In healthy tissue, intergenic and repetitive elements such as retrotransposons are typically methylated, contributing to chromatin compaction and genomic integrity. In malignancy, however, global hypomethylation of these regions is common and has been linked to chromatin decondensation, genomic instability, and aberrant transcriptional activity. Loss of imprinting control and activation of usually silenced elements, such as LINE-1 retrotransposons, are implicated in oncogenesis through enhanced mutational burden and genomic rearrangements. By contrast, CpG islands in promoter regions of essential genes are generally unmethylated under normal physiological conditions, ensuring chromatin accessibility and active gene transcription. Aberrant hypermethylation of these promoter CpG islands, leading to gene silencing, is among the most frequent somatic epigenetic alterations in cancer [103,104,105,106].

Histone modifications represent another central axis of epigenetic regulation. Histone tails, primarily at the N-terminus, undergo diverse post-translational modifications, including methylation, acetylation, ubiquitylation, phosphorylation, and ribosylation, which influence chromatin structure and gene accessibility. The functional outcome of these modifications is highly context-dependent, with gene expression governed not by individual marks in isolation but by the combinatorial “histone code” shaped by both the type and the site of modification. Histone methylation generally promotes chromatin condensation and transcriptional repression, whereas histone acetylation, catalysed by histone acetyltransferases and reversed by histone deacetylases, is typically associated with chromatin relaxation and gene activation. Notably, histone deacetylases also regulate non-histone substrates such as transcription factors and DNA repair enzymes, further extending their influence on cancer-relevant pathways. Knowing this is important since certain UPF-associated additives, such as emulsifiers and synthetic preservatives, seem to modulate histone acetylation and methylation patterns, thereby influencing gene expression in pathways implicated in tumour initiation [107,108,109].

Small non-coding RNAs, including miRNAs, piRNAs, siRNAs, and small nucleolar RNAs, represent an additional epigenetic layer of gene regulation. Though not translated into proteins, these RNAs modulate gene expression through mechanisms such as heterochromatin formation and translational inhibition and are implicated in regulating up to 60% of protein-coding genes. Their expression is under epigenetic control, with promoter hypermethylation and histone deacetylation contributing to miRNA silencing. Dysregulated miRNA expression, through either epigenetic silencing or functional perturbation, has been implicated in tumour initiation and progression, underscoring its potential role in maintaining genomic surveillance and homeostasis [110,111,112]. For instance, it seems that UPF-derived xenobiotics can alter miRNA expression profiles, thereby disrupting gene regulatory networks implicated in oncogenesis. Compounds such as plasticisers, commonly leached from UPF packaging or introduced during processing, as well as polycyclic aromatic hydrocarbons (PAHs) generated during high-temperature processing, have been shown to deregulate oncogenic microRNAs. These findings underscore the capacity of UPF constituents to reprogram cellular function through miRNA-mediated epigenetic mechanisms [113]. By inducing stable, heritable changes to gene expression, ultra-processed foods may silently reprogram the epigenome, tilting cellular physiology toward malignancy. These insights compel a shift in cancer prevention paradigms beyond caloric metrics and nutritional labels toward policy frameworks that explicitly consider the molecular legacy of modern diets.

Meta-analysis results of epigenome-wide association studies (EWASs) in European children showed associations that were only suggestive between total UPF intake and methylation of DNA at seven CpG sites; among these CpG sites are loci related to thyroid hormone signalling and hepatic metabolism [114]. However, this finding did not withstand the correction for multiple testing because it is likely that while UPFs may exert subtle epigenetic effects, the magnitude is unclear, as is its clinical relevance.

Another in vivo study in adolescents shows that high UPF consumption is significantly associated with higher urinary concentrations of 8-hydroxy-2′-deoxyguanosine (8-OHdG), a well-recognised biomarker of oxidative DNA damage [115]. This provides further support for the hypothesis that UPFs act through oxidative stress-related mechanisms to induce genomic instability. Other reviews of the literature also indicate that related UPF exposures—additives, contaminants from packaging, and nutrient imbalances—can influence DNA damage and epigenetic regulation through a range of converging mechanisms [116].

Current epidemiological data consider and evaluate UPF exposure as a global dietary pattern, characterised in most cases by the NOVA system classification, without distinction in specific food subtypes. Thus, existing in vivo human studies do not support, at this time, distinct epigenetic effects attributable to particular categories of UPFs [115].

In vitro studies have attempted to uncover the mechanistic bases by examining separate components of UPFs, such as emulsifiers, sweeteners, and advanced glycation end products (AGEs). Although these studies demonstrate that such compounds can alter patterns of DNA methylation and histone post-translational modifications in cell lines, they are accompanied by several significant limitations. Above all else, the in vitro model is unable to fully replicate or represent the actual complexity of digestion, absorption, and metabolism, or host–microbiome interactions, both of which are crucial for modifying the biological activity of dietary components [117].

Moreover, it is nearly impossible to simulate in vitro the complexity of the food matrix in UPFs—the result of multiple additives and processing by-products, as well as altered nutrient ratios—which hampers the isolation of causal agents and the generalisation of findings to real-world exposures [118]. The systems also focus on short-term high-dose exposures, whereas in vivo long-term low-level exposures are probably more relevant to epigenetic reprogramming leading to chronic disease, and such conditions are not easily mimicked in vitro [119].

The above mechanisms are plausible from in vitro studies, and associations have been reported in observational human studies. However, in vivo experimental validation is missing. Thus, future research trends should be directed toward merging controlled animal studies with human biomarker data to bridge this translational gap and define the causal relationship between UPF exposure and epigenetic remodelling.

### 6.3. Low-Grade Immune Activation in UPF-Driven Carcinogenesis

**Figure 4:**
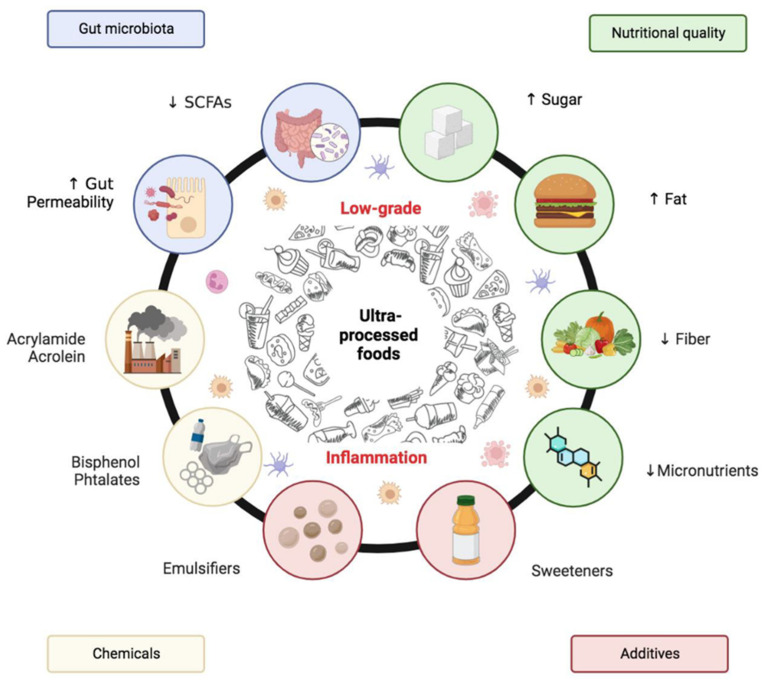
UPF and low-grade inflammation relationship. Reproduced with permission from Nutrients; published by MDPI [124].

Chronic low-grade inflammation, as persistent and subclinical immune system activation, has been more significantly causally connected in recent times with UPF consumption and cancer development, most notably in young populations. In contrast to acute inflammation, a short-lived protective response to tissue injury or infection, low-grade inflammation is a more insidious and pervasive condition that is distributed throughout the human body. It consists of prolonged increases in proinflammatory mediators such as C-reactive protein (CRP), interleukin-6 (IL-6), and tumour necrosis factor-alpha (TNF-α). These mediators help drive cancer development by activating oxidative stress, leading to DNA damage and epigenetic changes, blocking apoptosis, and, in many ways, fostering immune evasion [120,121]. Emerging evidence suggests that UPFs do more than provide nutrients in an imbalanced manner; they also act as active biological agents that initiate and maintain low-grade inflammation (Figure 4).

This is partly mediated by the nonnutritive components (artificial sweeteners, emulsifiers, colourants, and preservatives) that disturb the balance of gut microbes and increase gut lining permeability, triggering systemic immune activation. For example, in animal models, it has been shown that artificial sweeteners, such as aspartame and sucralose, can influence inflammatory and hormonal signalling. Aspartame consumption in rats causes significant alterations in thyroid hormone levels and increases both anti-inflammatory and pro-inflammatory cytokines. It presents a dual disruption of hormonal and immune regulation. Sucralose has been reported to cause insulin resistance and systemic inflammation, probably through its action on gut microbiota [122,123].

In humans, it has been reported that non-nutritive sweeteners (NNSs) cause changes in metabolic parameters and gut microbial profiles, which then play a significant role in systemic inflammation. Sylvetsky et al. reported correlations between the consumption of NNSs and inflammatory biomarkers (CRP and IL-6), primarily in individuals with an elevated body mass index (BMI) [125]. From these results, it can be hypothesised that sweeteners may enhance inflammation in metabolically at-risk populations, thereby promoting systemic effects leading to cancer. Additionally, the dietary context appears to be very important; when combined with saturated fats, some sweeteners have been shown to exacerbate metabolic endotoxemia, thereby increasing inflammatory signalling and metabolic dysfunction [126].

The young population is very vulnerable to proinflammatory and cancer-causing effects linked to UPF consumption. During childhood and adolescence, periods of active immune system development and epigenetic programming render these systems more susceptible to chronic inflammatory damage. Epidemiological studies have shown that teenagers with high UPF consumption exhibit increased levels of inflammatory mediators such as CRP, IL-6, and TNF-α [127]. In turn, these biomarkers are active components in inflammation, autoimmune imbalance, and the impairment of genomic protection and cellular restoration mechanisms. DNA damage targeted by inflammation is mediated through reactive oxygen and nitrogen species produced by immune cells, which in turn lead to mutations and genomic instability. This chronically inflamed microenvironment may further support the survival of premalignant clones, facilitated by suppressed apoptosis and stimulated proliferation [128].

Diet-induced low-grade inflammation has been linked with the risk of cancer in several population-based studies. The proinflammatory dietary pattern during adolescence was leading to premalignancy of breast cancer, which was identified by Harris et al. [129]. Also, systemic inflammation indicated by CRP has been connected with colorectal, lung, and breast cancer development [130]. These links are concerning in AYAs since early-life diet exposures may have lasting cancer-causing effects through building up harmful immune and metabolic disruptions.

UPFs significantly increase the risk of obesity and metabolic syndrome, conditions associated with chronic inflammation and cancer. Obese people tend to have raised levels of adipokines and inflammatory mediators produced by fat cells, thus creating a body-wide condition that forces cancer initiation and progression. In this respect, the dietary inflammatory index (DII) and other inflammation-linked diet scoring schemes have shown that diets that cause inflammation, especially UPFs, are associated with worse cancer incidence and prognostic outcomes [124,131].

Additionally, the inflammation triggered by UPFs may gain additional traction through epigenetic changes, especially in adolescent cancers. Chronic inflammation can disrupt the normal processes of DNA methylation and histone modification. This leads not only to the silencing of tumour suppressor genes, such as CDKN2A and MGMT, but also to the activation of oncogenic pathways. Paediatric tumours, such as rhabdomyosarcoma, acute lymphoblastic leukaemia, and brain tumours, often exhibit fusion genes, chromosome breaks and holes, and histone mutations, many of which may be caused or maintained by long-term exposure to inflammation. Inflammatory cytokines such as TNF-α and IL-6 have been shown to upregulate histone demethylases, including Jmjd3 (KDM6B), which affects the expression of genes, leading to stem-like, therapy-resistant tumour phenotypes [120].

In summary, the combination of UPF consumption, low-grade inflammation, and the increased immune weakness of adolescents shapes a straightforward way to understand early-onset cancer formation. UPFs act as “silent disrupters” of immune and metabolic balance, thereby creating a biological environment that facilitates cancer transformation long before the visible signs of illness become apparent.

In vitro models comprising human intestinal epithelial cell lines (Caco-2, HT29, T84) and immune cell lines (THP-1, RAW 264.7) have demonstrated that exposure to common UPF components leads to the upregulation of pro-inflammatory cytokines (IL-6, IL-8, TNF-α), oxidative stress, and damage to tight junctions. Emulsifiers such as carboxymethylcellulose and polysorbate 80 can induce inflammatory responses when tested in various models. Results from processed food digest (e.g., white chocolate or sausage) provide further evidence. Moreover, co-culture and tri-culture systems exacerbate these effects, thereby demonstrating epithelial–immune cross-talk [132,133,134].

3D gut models and organoid-derived monolayers from IBD patients exhibit sensitised inflammatory responsiveness to food emulsifiers such as carrageenan, characterised by increased cytokine expression and subsequent barrier disruption. Such physiologically relevant platforms were utilised to investigate the early immune activation elicited by UPF components. The in vitro findings of emulsifiers, artificial sweeteners, and formulations of the Western diet have been confirmed in rodent models; chronic exposure to these compounds results in increased intestinal permeability, activation of NF-κB, and inflammation within the mucosa. Mice given UPF-rich diets exhibit significantly higher levels of TNF-α, IL-6, and iNOS expression, along with metabolic disease features such as insulin resistance, adiposity, and dyslipidaemia [135].

Importantly, susceptibility to UPF-induced inflammation is influenced by host genetics and baseline immune status. Thus, rodents with IL-10 deficiency or pathobiont colonisation by adherent–invasive *E. coli* exhibit sensitised proinflammatory responses to emulsifiers in a Western dietary pattern [136]. This also aligns with human observational studies; stronger associations have been reported between UPF intake and biomarkers of inflammation (e.g., CRP, INFLA-score) in individuals with obesity, metabolic syndrome, or older age [101,137].

Taken together, the in vitro, in vivo, and epidemiological evidence converges on the fact that UPF components play a role in driving and maintaining chronic low-grade inflammation, a central permissive factor in carcinogenesis and other chronic pathologies. While the available in vitro data have demonstrated mechanistic plausibility and animal models’ causality support, future translational studies should advance actionable definitions of dose–response relationships and at-risk populations among humans.

## 7. UPFs as a Trojan Horse for Cancer Development

All the previously mentioned mechanisms produce silent, quantitatively measurable damage accumulated over time without noticeable symptoms or biochemical markers, illustrating the Trojan horse model. In this proposed model, UPFs produce immediate toxicity; instead, they act through biological mimicry, slowly disrupting the balance of cell health and evading conventional methods of prevention. UPFs should no longer be seen as a source of low-nutritive-value calories in clinical settings. Instead, they should be regarded as complex biological agents whose effects are not acute but rather are quiet and accumulate in the long run by disrupting the body’s critical regulatory systems [138]. Classical dietary paradigms, which centre on macronutrients and caloric balance, cannot explain the overall health effects of ultra-processed foods. From this point of view, the Trojan horse concept provides a clear framework for understanding how UPFs disrupt physiological balance from the inside, whereby the body cannot often recognise threats. The key parts of the Trojan horse model that act as a way to understand the tricky effects of UPFs via endocrine-disrupting chemicals include biological masking (not being seen as a danger), disruption in many ways (affecting hormonal, immune, microbial, and gene-setting systems), quiet and building harm quietly and with no immediate warning signs, and their mind-affecting impact that leads to forced eating by changing how the brain’s reward system works.

A fundamental trait of UPFs as a Trojan horse lies in their ability to deliver to the body a wide range of substances and mechanisms of action that are not toxic by classical criteria but whose synergy despoils several highly tuned systems usually involved in controlling growth, differentiation, inflammatory responses, metabolic homeostasis, and cell cycling [139]. This concept applies clinically because these derangements occur silently, without overt acute laboratory manifestations, and are often beyond the capacities of routine diagnostic algorithms. However, their gradual accumulation eventually leads to a functional breakdown in the mechanisms that control proliferation and regeneration, thus creating an environment that promotes carcinogenic changes [140].

UPFs do not work as a primary carcinogen in classical causation; they do not cause cancer directly the way tobacco smoke or ionising radiation does. They act biologically as a catalyst of disturbance, gradually removing the defence mechanisms. Their components do not have any structure the body recognises as dangerous, such as chemicals from packaging and additives, broken dietary signals, and organic nutritional molecules replaced by synthetic and non-informative compounds. Due to this ability to mimic, they circumvent innate sensory and immune barriers [141].

UPFs contribute to the process of carcinogenesis not only through an imbalance of nutrients but also through several other mechanisms. These include disturbances in lipid metabolism, the dysregulation of adipokines, low-grade chronic inflammation, and changes in the extracellular matrix. High-UPF-consumption diets have been shown to cause immune dysfunction, increase the expression of proinflammatory cytokines such as TNF-α and IL-6, and simultaneously alter hormonal profiles and destabilise gut microbiota [140].

From a functional perspective, UPFs can be viewed as metabolic and informational toxicants that do not directly kill cells but damage their environmental perception. They disrupt the hormonal, immunomodulatory, and microbiome metabolite signalling pathways and alter the epigenetic record without modifying the DNA sequence, resulting in subclinical effects on tissue regeneration and cellular differentiation [142]. This is why their action can be likened to that of a false programmer; they do not wipe out the genetic code but instead rewrite it subtly and gradually.

Recent work by Lustig further supports conceptualising UPFs as the “Trojan horse” of biological dysfunction, meaning that UPFs meet every criterion for public health regulation in toxicity, abuse, ubiquity, and harmful externalities [143]. The author, therefore, highlights that the main ingredients of UPFs, one being high-fructose corn syrup, represent more than just a caloric surplus; instead, their direct metabolic effects include lipogenesis de novo in the liver, generation of reactive oxygen species, and stimulation of signalling pathways related to inflammation, insulin resistance, and epigenetic change. Fructose differs significantly from glucose in its action, as it does not stimulate insulin and leptin secretion. Still, it appears to act only through the reward pathway, leading to desensitisation of D2-type dopamine receptors, which causes reductions in self-control via suppression mechanisms in the prefrontal cortex. This pattern falls into the neurobiological category, similar to that of psychoactive substances. The same author further remarks that UPFs are not a neutral food. They are an agent that behaves like a pharmacologically active substance and acts quietly cumulatively. The changes not only lead to metabolic syndrome and liver disease but also hold long-lasting alterations in gene expression, oxidative balance, and neuroendocrine response; therefore, homeostasis becomes compromised in the long term and, thus, leads to an increased risk of several chronic diseases, including cancer. What is more alarming is that these impacts are now visible in children wherein increasingly common cases of non-alcoholic fatty liver disease and type 2 diabetes are being diagnosed; this means UPFs work from the earliest stages of life quietly yet systematically, hence establishing their identification as a modern Trojan agent.

The Trojan mechanism of UPFs does not act in a single spot or at a single moment in time. It acts simultaneously on three primary biological levels: signalling (endocrine), communication (macrobiotic), and epigenetic (regulatory). These three layers form invisible perpetrators within a dietary pattern that seems innocuous but comprises factors that actively destabilise cellular homeostasis. Simultaneously, the mechanisms involved are not linear; they are interconnected, synchronised, and self-amplifying, such that multi-channel disruption leads to a loss of control over cellular proliferation and immune surveillance.

This model does not initiate carcinogenesis through mutation. Instead, it initiates carcinogenesis through communication disruption and the loss of valid information between the host and its microbiological, metabolic, and hormonal environment. Since the body does not recognise the dietary component as the cause, this process does not set off alarm signals. The ultimate result is equivalent to “bioinformational noise” conditions in which cells can no longer distinguish accurate signals from spurious ones. In the long term, a cellular identity will develop that no longer supports differentiation pathways but instead adopts proliferative, proinflammatory, and hypoxic behaviour corresponding to the early-stage tumour microenvironment [144].

The model’s validity proved rather optimistic when applied to the colorectal cancer case. The intestinal mucosa has long been known as one of the most metabolically active tissues, with a very high cell turnover and constant contact between luminal content and cells. Should UPFs disrupt microbiomes, compromise barrier functions, and affect the epigenetic stability of intestinal cells, the colorectal epithelium logically becomes the first site of clinical manifestation [145].

The classical approaches to risk assessment, such as dose–response, LD50, and acute toxicity, do not provide a strategy for identifying slow-progressing, multifactorial mechanisms. UPFs cause cumulative systemic shifts that are difficult to measure and not picked up by classical tests. However, they leave numerous and long-lasting perturbations in body control systems. This calls for a new definition of “food safety” and a broader focus than nutritional value on the biological effects of diet in general [146].

**Figure 5:**
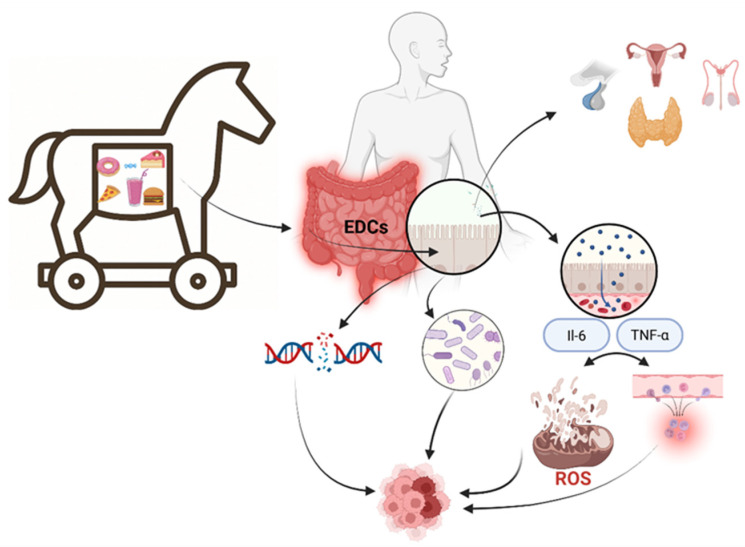
The Trojan horse mechanism: how ultra-processed foods introduce endocrine disruptors and promote cancer-related pathways. Created with Biorender.com (accessed on 20 May 2025). License obtained.

UPFs include industrial formulations that often contain additives, such as emulsifiers, artificial sweeteners, colours, and stabilisers, that produce effects on gut microbiota. They may thin mucosal layers and enhance permeability; this is followed by systemic inflammation [147]. Simultaneously, plastic UPF product packaging often contains contaminants that easily migrate into foods and thus supply endocrine-disrupting activity. Compounds belong to the nutritional component but reach the internal system through external contamination (packaging) and thereby cause hormonal imbalance, oxidative stress, and neoplastic transformation through epigenetic changes (Figure 5). Other industrial processing results in harmful baked and dried byproducts such as acrylamide and acrolein, known genotoxins and proinflammatory agents.

Furthermore, UPFs are elaborated to deliver enormous amounts of sugar, salt, and fat along with flavour enhancers, making attractive products one would like to consume repeatedly, leading to compulsive consumption and addictive behaviour, hence allowing UPFs as an agent not only to infiltrate the body via metabolic pathway hormonal passage but also through the central nervous system, altering reward centres that regulate behaviour [148]. Thus, UPFs becomes more than a dietary pattern. They become a platform for simultaneous multi-channel interference, placing metabolic, endocrine, immune, epigenetic, and neuropsychological spheres under attack. This multi-layered, gradual, hard-to-detect nature of action would strengthen the metaphor of the Trojan horse: the organism does not recognise the harm until it has already penetrated deeply into its regulatory structures [38].

Besides their known metabolic and inflammatory effects, UPFs have emerged with their microstructural and sensory-manipulative properties as additional layers of “biological disguise.” Tobias and Hall argue that UPFs are designed to require minimal chewing or swallowing and swift energy intake, thereby bypassing the slower-acting mechanisms of the gut–brain axis and leading to overeating, as satiety signals do not catch up. Furthermore, industrial processing removes water and fibre from these products; thus, energy density is added to them, accompanied by a simultaneous reduction in mechanical and metabolic resistance. Engineered properties devoid of any evolutionarily relevant nutritional signalling make UPFs essentially “invisible” to intrinsic body appetite- and satiety-sensing regulatory systems [149].

The UPF–Trojan horse conceptual model argues that UPFs act not as direct carcinogens but as insidious disruptors of the informational networks that stabilise cellular identity and immune surveillance. The Trojan horse metaphor underscores the complex and often unrecognisable nature of these effects, which are silent, cumulative, and at the core of a globally prevalent dietary pattern. The impacts of UPFs involve a variety of mechanisms, ranging from endocrine disruption to destabilisation of the microbiome, epigenetic changes, and neurobehavioral manipulation, which impose system-wide stress resembling pharmacological and toxicological pathways outside traditional risk assessment frameworks. This sets an agenda for another paradigm shift in health-impact food evaluation, moving beyond nutrients toward total biological effects. However, despite the growing evidence in favour of this concept, very critical questions remain unanswered. To move the field forward, gaps in epidemiological consistency, mechanistic validation, and long-term causality must be identified and addressed.

## 8. Knowledge Gap and Future Directions

Despite growing concern over the role of UPFs in cancer development, several critical knowledge gaps persist. Epidemiological studies examining the association between UPF consumption and cancer risk face several common confounding factors that can impact the validity of their findings. Lifestyle factors are significant confounders in these studies, and variables such as BMI, physical activity, smoking, alcohol consumption, and overall dietary patterns can influence cancer risk and are often correlated with UPF consumption. For instance, the study by Wang et al. adjusted for BMI and dietary quality, yet residual confounding from unmeasured lifestyle factors may still affect the results [150]. Similarly, Jafari et al. adjusted for BMI, income, smoking, type of job, educational level, and physical activity, but the potential for residual confounding remains [151].

Misclassification of food items is another critical limitation. The NOVA classification system, which is commonly used to define UPFs, may not always accurately categorise foods, leading to misclassification bias. This issue is highlighted in the systematic review by Isaksen et al., which noted the likely misclassification of several foods as UPFs or non-UPFs in multiple studies [99]. Accurate classification is essential to ensure the reliability of dietary assessments and subsequent risk estimations.

Gender differences in health outcomes also present a challenge. The study by Wang et al. found a significant association between UPF consumption and colorectal cancer risk in men but not in women, suggesting potential gender-specific effects [150]. Similarly, Zhong et al. reported that the harmful associations of UPF consumption with cardiovascular mortality were more pronounced in women, indicating that gender may modify the relationship between UPF intake and health outcomes [152]. These differences necessitate further investigation into the underlying biological or lifestyle factors contributing to these disparities.

Overall, while there is suggestive evidence linking UPF consumption to increased cancer risk, and these confounding factors underscore the need for a careful study design and analysis to assess the relationship between UPF consumption and cancer risk accurately. Future research should aim to improve dietary assessment methods, account for potential confounders more comprehensively, and explore gender-specific effects to clarify these associations.

The need for multi-omics and longitudinal studies arises from the limitations of current epidemiological research, which often relies on observational data that can be confounded by various factors such as lifestyle and dietary patterns. Multi-omics approaches, which integrate genomics, proteomics, metabolomics, and other omics data, can provide a more comprehensive understanding of the biological mechanisms underlying the association between UPF consumption and cancer risk. These studies can help identify specific biomarkers and pathways involved in carcinogenesis related to UPF intake.

Longitudinal studies are crucial for establishing temporal relationships and causality, as they track dietary habits and health outcomes over time. They can help address issues of reverse causation and provide insights into the long-term effects of UPF consumption on cancer risk. Additionally, they can account for changes in dietary patterns and other lifestyle factors that may influence cancer risk.

## 9. Conclusions

UPFs have been implicated as silent drivers of metabolic, endocrine, and oncogenic disruption in modern populations, particularly among AYAs. Through multiple converging pathways, ranging from microbiome dysbiosis and epigenetic reprogramming to chronic inflammation and endocrine disruption, UPFs may contribute to a biologically permissive environment for tumour initiation and progression. These effects are often subtle and cumulative, occurring without overt biochemical warning signs, which reinforces the concept of UPFs as modern “Trojan horses” of human physiology. This review highlights the inadequacy of classical dietary risk models, which focus solely on caloric and macronutrient content. It proposes a paradigm shift toward recognising the complex, systemic impacts of industrially processed diets. Given the alarming rise in early-onset cancers and the disproportionate consumption of UPFs in younger populations, immediate attention from both the scientific and public health communities is warranted. In addition, recognising the heterogeneity of responses to UPF exposure, shaped by genetic background, microbiome composition, and epigenetic variability, emphasises the need for personalised nutrition and precision medicine approaches. Integrating molecular profiling, dietary assessment, and individual risk stratification may enable the development of tailored interventions that more effectively mitigate the carcinogenic potential of UPFs.

## Figures and Tables

**Table 1:** Dietary energy derived from UPFs in youth populations.

Author (Year)	Country	Sample Size	Age Group (Years)	UPF (%)
Rocha et al. (2021) [13]	Brazil	71,533	12–17	28.0
Oliveira et al. (2021) [14]	US	462	13.1 ± 1.5	31.9
Martinez Steele et al. (2020) [15]	Brazil	9416	12–19	66.9
Sousa et al. (2020) [16]	Brazil	2499	18–19	35.8
Viola et al. (2020) [17]	Brazil	1525	18–19	37.0
Enes et al. (2019) [18]	Brazil	200	10–18	50.6
Melo et al. (2021) [19]	Brazil	804	16.1 ± 1.2	45.9
D’Avila et al. (2017) [20]	Brazil	784	15.2 ± 1.3	49.2
Rauber et al. (2021) [21]	UK	542	11–18	67.8
Wanjohi et al. (2025) [22]	Kenya	621	10–19	25.2

**Table 2:** Microbial and metabolic biomarkers linking UPFs, dysbiosis, and CRC.

Biomarker	Microbial Source	Host Effect	Role in CRC
Butyrate	*Faecalibacterium*, *Roseburia* spp.	Energy for colonocytes, HDAC inhibition, anti-inflammatory	Deficiency promotes epigenetic instability and inflammation [77,78]
Propionate	*Bacteroides*, *Veillonella* spp.	Anti-inflammatory, maintains the mucosal barrier	Reduction impairs immune regulation [77]
Hydrogen sulphide	*Desulfovibrio* spp.	Mitochondrial damage, oxidative stress, stem cell disruption	Genotoxic and proinflammatory [82]
DCA, LCA	*Clostridium*, *Bacteroides* spp.	DNA damage, oxidative stress, pathway activation	Promote Wnt/β-catenin, EGFR signalling [83]
TMAO	*Microbial metabolism of choline*/*carnitine*	Angiogenesis, cell proliferation via oncogenic signalling	Promotes tumour progression [84]
LPS	*Gram-negative bacteria*	Activates TLR4 and NF-κB, induces IL-6 and TNF-α	Drives chronic inflammation and cancer progression [85]

## Data Availability

No new data were created or analysed in this study. Data sharing is not applicable to this article.

## Abbreviations

AHR	Aryl Hydrocarbon Receptor
ASIR	Age-Standardised Incidence Rate
ASMR	Age-Standardised Mortality Rate
AYAs	Adolescents and Young Adults
BPA	Bisphenol A
CRC	Colorectal Cancer
CRP	C-Reactive Protein
CpG	Cytosine–phosphate–Guanine
DCA	Deoxycholic Acid
DEHP	Di(2-ethylhexyl) Phthalate
DINP	Diisononyl Phthalate
DII	Dietary Inflammatory Index
DNA	Deoxyribonucleic Acid
EDCs	Endocrine-Disrupting Chemicals
EMT	Epithelial–Mesenchymal Transition
EPA	Eicosapentaenoic Acid (if referenced)
GM	Gut Microbiota
HDAC	Histone Deacetylase
IL-6	Interleukin-6
LCA	Lithocholic Acid
LPS	Lipopolysaccharide
NNS	Non-Nutritive Sweeteners
NOVA	Not an acronym—name of food classification system
PAHs	Polycyclic Aromatic Hydrocarbons
SCFA	Short-Chain Fatty Acids
TLR4	Toll-Like Receptor 4
TMAO	Trimethylamine N-oxide
TNF-α	Tumour Necrosis Factor-alpha
UPF	Ultra-Processed Food
UPP	Ultra-Processed Product

## References

[1] Gibney M.J. (2018). Ultra-processed foods: Definitions and policy issues. Curr. Dev. Nutr..

[2] Capozzi F., Magkos F., Fava F., Milani G.P., Agostoni C., Astrup A., Saguy I.S. (2021). A multidisciplinary perspective of ultra-processed foods and associated food processing technologies: A view of the sustainable road ahead. Nutrients.

[3] Henney A.E., Gillespie C.S., Alam U., Hydes T.J., Boyland E., Cuthbertson D.J. (2024). Ultra-processed food and non-communicable diseases in the United Kingdom: A narrative review and thematic synthesis of literature. Obes. Rev..

[4] Paramasivam A., Murugan R., Jeraud M., Dakkumadugula A., Periyasamy R., Arjunan S. (2024). Additives in processed foods as a potential source of endocrine-disrupting chemicals: A review. J. Xenobiot..

[5] Rondinella D., Raoul P.C., Valeriani E., Proietti S., Scazzocchio B., Masella R. (2025). The detrimental impact of ultra-processed foods on the human gut microbiome and gut barrier. Nutrients.

[6] Bhatia S., Pappo A.S., Acquazzino M., Allen-Rhoades W.A., Barnett M., Borinstein S.C., Casey R., Choo S., Chugh R., Dinner S. (2023). Adolescent and Young Adult (AYA) Oncology, Version 2.2024, NCCN Clinical Practice Guidelines in Oncology. J. Natl. Compr. Canc. Netw..

[7] Hughes T., Harper A., Gupta S., Frazier A.L., van der Graaf W.T.A., Moreno F., Joseph A., Fidler-Benaoudia M.M. (2024). The current and future global burden of cancer among adolescents and young adults: A population-based study. Lancet Oncol..

[8] Li J., Kuang X. (2024). Global cancer statistics of young adults and its changes in the past decade: Incidence and mortality from GLOBOCAN 2022. Public Health.

[9] di Martino E., Smith L., Bradley S.H., Hemphill S., Wright J., Renzi C., Bergin R., Emery J., Neal R.D. (2022). Incidence trends for twelve cancers in younger adults—A rapid review. Br. J. Cancer.

[10] Toss A., Piombino C., Quarello P., Trama A., Mascarin M., Lambertini M., Canesi M., Incorvaia L., Milano G.M., Maruzzo M. (2024). Risk factors behind the increase of early-onset cancer in Italian adolescents and young adults: An investigation from the Italian AYA Working Group. Eur. J. Cancer.

[11] Lord S.R., Harris A.L. (2023). Is it still worth pursuing the repurposing of metformin as a cancer therapeutic?. Br. J. Cancer.

[12] Le N.T., Pham Y.T.-H., Le L.T., Dao H.V., Koriyama C., Ha T.H., Lichtveld M., Kuchipudi S.V., Huynh N.Y.-N., Nguyen D.D. (2024). Factors affecting cancer mortality in young adults: Findings from a prospective cohort study. Cancers.

[13] Rocha L.L., Gratão L.H.A., do Carmo A.S., Costa A.B.P., de Freitas Cunha C., de Oliveira T.R.P.R., Mendes L.L. (2021). School Type, Eating Habits, and Screen Time are Associated with Ultra-Processed Food Consumption among Brazilian Adolescents. J. Acad. Nutr. Diet..

[14] Oliveira R.R., Peter N.B., Muniz L.C. (2021). Food consumption according to the level of processing among adolescents from the rural area of a municipality in the south of Brazil. Cienc. Saude Coletiva.

[15] Martínez Steele E., Khandpur N., da Costa Louzada M.L., Monteiro C.A. (2020). Association between dietary contribution of ultra-processed foods and urinary concentrations of phthalates and bisphenol in a nationally representative sample of the US population aged 6 years and older. PLoS ONE.

[16] Sousa R.D.S., Bragança M.L.B.M., Oliveira B.R.D., Coelho C.C.N.D.S., Silva A.A.M.D. (2020). Association between the Degree of Processing of Consumed Foods and Sleep Quality in Adolescents. Nutrients.

[17] Viola P.C.D.A.F., de Carvalho C.A., Bragança M.L.B.M., da Cunha França A.K.T., de Britto M.T.S.S., da Silva A.A.M. (2020). High consumption of ultra-processed foods is associated with lower muscle mass in Brazilian adolescents in the RPS birth cohort. Nutrition.

[18] Enes C.C., Camargo C.M.D., Justino M.I.C. (2019). Ultra-processed food consumption and obesity in adolescents. Rev. Nutr..

[19] Melo A.S., Neves F.S., Batista A.P., Machado-Coelho G.L.L., Sartorelli D.S., de Faria E.R., Netto M.P., Oliveira R.M., Fontes V.S., Cândido A.P.C. (2021). Percentage of energy contribution according to the degree of industrial food processing and associated factors in adolescents (EVA-JF study, Brazil). Public Health Nutr..

[20] D’avila H.F., Kirsten V.R. (2017). Energy intake from ultra-processed foods among adolescents. Rev. Paul. Pediatr..

[21] Rauber F., Martins C.A., Azeredo C.M., Leffa P.S., da Costa Louzada M.L., Levy R.B. (2022). Eating context and ultraprocessed food consumption among UK adolescents. Br. J. Nutr..

[22] Wanjohi M.N., Asiki G., Wilunda C., Holdsworth M., Pradeilles R., Paulo L.S., Langat N., Amugsi D.A., Kimenju S., Kimani-Murage E.W. (2025). Ultra-processed food consumption is associated with poor diet quality and nutrient intake among adolescents in urban slums, Kenya. Int. J. Public Health.

[23] Chang K., Gunter M.J., Rauber F., Millett C., Imamura F., Shi Z., Scarborough P., Monteiro C.A., Forouhi N.G. (2023). Ultra-processed food consumption, cancer risk and cancer mortality: A large-scale prospective analysis within the UK Biobank. EClinicalMedicine.

[24] Aramburu A., Alvarado-Gamarra G., Cornejo R., Curi-Quinto K., Díaz-Parra C.D.P., Rojas- Limache G., Lanata C.F. (2024). Ultra-processed foods consumption and health-related outcomes: A systematic review of randomised controlled trials. Front. Nutr..

[25] Fardet A. (2024). Ultra-processing should be understood as a holistic issue, from food matrix, to dietary patterns, food scoring, and food systems. J. Food Sci..

[26] Monteiro C.A., Cannon G., Levy R.B., Moubarac J.-C., Louzada M.L.C., Rauber F., Khandpur N., Cediel G., Neri D., Martinez-Steele E. (2019). Ultra-processed foods: What they are and how to identify them. Public Health Nutr..

[27] Braesco V., Souchon I., Sauvant P., Haurogné T., Maillot M., Féart C., Darmon N. (2022). Ultra-processed foods: How functional is the NOVA system?. Eur. J. Clin. Nutr..

[28] Egnell M., Kesse-Guyot E., Galan P., Touvier M., Rayner M., Jewell J., Breda J., Hercberg S., Julia C. (2018). Impact of Front-of-Pack Nutrition Labels on Portion Size Selection: An Experimental Study in a French Cohort. Nutrients.

[29] McClements D.J. (2023). Ultraprocessed plant-based foods: Designing the next generation of healthy and sustainable alternatives to animal-based foods. Compr. Rev. Food Sci. Food Saf..

[30] Levy R.B., Barata M.F., Leite M.A., Andrade G.C. (2024). How and why ultra-processed foods harm human health. Proc. Nutr. Soc..

[31] Hafner E., Hribar M., Pravst I. (2025). Ultra-Processed Foods in the Food Supply: Prevalence, Nutritional Composition and Use of Voluntary Labelling Schemes. Nutrients.

[32] Viennois E., Merlin D., Gewirtz A.T., Chassaing B. (2017). Dietary Emulsifier-Induced Low-Grade Inflammation Promotes Colon Carcinogenesis. Cancer Res..

[33] Dai S., Wellens J., Yang N., Li D., Wang J., Wang L., Yuan S., He Y., Song P., Munger R. (2024). Ultra-processed foods and human health: An umbrella review and updated meta-analyses of observational evidence. Clin. Nutr..

[34] Robinson E., Cummings J.R., Gough T., Jones A., Evans R. (2024). Consumer Awareness, Perceptions and Avoidance of Ultra-Processed Foods: A Study of UK Adults in 2024. Foods.

[35] Juul F., Bere E. (2024). Ultra-processed foods—A scoping review for Nordic Nutrition Recommendations 2023. Food Nutr Res..

[36] Vignesh A., Amal T.C., Vasanth K. (2024). Food contaminants: Impact of food processing, challenges and mitigation strategies for food security. Food Res. Int..

[37] Ubbink J., Levine A.S. (2025). From Processed Foods to Ultraprocessed Foods: Evolution of an Industry Model and Impact on Dietary Quality, Health, and Society. Annu. Rev. Food Sci. Technol..

[38] Esposito S., Gialluisi A., Di Castelnuovo A., Costanzo S., Pepe A., Ruggiero E., De Curtis A., Persichillo M., Cerletti C., Donati M.B. (2024). Ultra-processed food consumption is associated with the acceleration of biological aging in the Moli-sani Study. Am. J. Clin. Nutr..

[39] Melough M.M., Maffini M.V., Otten J.J., Sathyanarayana S. (2022). Diet quality and exposure to endocrine-disrupting chemicals among US adults. Env. Res..

[40] Zhao H., Gui W., Liu S., Zhao F., Fan W., Jing F., Sun C. (2024). Ultra-processed foods intake and sex hormone levels among children and adolescents aged 6–19 years: A cross-sectional study. Front. Nutr..

[41] Oviedo-Solís C.I., Monterrubio-Flores E.A., Cediel G., Denova-Gutiérrez E., Barquera S. (2022). Trend of Ultraprocessed Product Intake Is Associated with the Double Burden of Malnutrition in Mexican Children and Adolescents. Nutrients.

[42] Pearce E.N. (2024). Endocrine Disruptors and Thyroid Health. Endocr. Pract..

[43] Lagarde F., Beausoleil C., Belcher S.M., Belzunces L.P., Emond C., Guerbet M., Rousselle C. (2015). Non-monotonic dose-response relationships and endocrine disruptors: A qualitative method of assessment. Environ. Health.

[44] Yilmaz B., Terekeci H., Sandal S., Kelestimur F. (2020). Endocrine disrupting chemicals: Exposure, effects on human health, mechanism of action, models for testing and strategies for prevention. Rev. Endocr. Metab. Disord..

[45] Hansel M.C., Rosenberg A.M., Kinkade C.W., Capurro C., Rivera-Núñez Z., Barrett E.S. (2024). Exposure to synthetic endocrine-disrupting chemicals in relation to maternal and fetal sex steroid hormones: A scoping review. Curr. Environ. Health Rep..

[46] Jalal N., Surendranath A.R., Pathak J.L., Yu S., Chung C.Y. (2017). Bisphenol A (BPA) the mighty and the mutagenic. Toxicol. Rep..

[47] Kurşunoğlu N.E., Sarer Yurekli B.P. (2022). Endocrine disruptor chemicals as obesogen and diabetogen: Clinical and mechanistic evidence. World J. Clin. Cases.

[48] Lugnier C., Meyer A., Charloux A., Andrès E., Gény B., Talha S. (2019). The endocrine function of the heart: Physiology and involvements of natriuretic peptides and cyclic nucleotide phosphodiesterases in heart failure. J. Clin. Med..

[49] Zeng B., Wu Y., Huang Y., Zhang Y., Li X., Liu Y., Wang Y., Chen L., Zhou Y., Tang S. (2024). Carcinogenic health outcomes associated with endocrine disrupting chemicals exposure in humans: A wide-scope analysis. J. Hazard. Mater..

[50] Kassotis C.D., Vandenberg L.N., Demeneix B.A., Porta M., Slama R., Trasande L. (2020). Endocrine-disrupting chemicals: Economic, regulatory, and policy implications. Lancet Diabetes Endocrinol..

[51] Buoso E., Masi M., Racchi M., Corsini E. (2020). Endocrine-disrupting chemicals’ (EDCs) effects on tumour microenvironment and cancer progression: Emerging contribution of RACK1. Int. J. Mol. Sci..

[52] Li W.P., Wang Y.F., Gao J., Yu M.L., Yu Y.Y., Yao Y.Q. (2014). In vitro evidence for endocrine-disrupting chemical (EDC)'s inhibition of drug metabolism. Afr. Health Sci..

[53] Sneha S., Baker S.C., Green A., Storr S., Aiyappa R., Martin S., Pors K. (2021). Intratumoural Cytochrome P450 Expression in Breast Cancer: Impact on Standard of Care Treatment and New Efforts to Develop Tumour-Selective Therapies. Biomedicines.

[54] Nebert D.W. (2017). Aryl hydrocarbon receptor (AHR): “Pioneer member” of the basic-helix/loop/helix per-Arnt-sim (bHLH/PAS) family of “sensors” of foreign and endogenous signals. Prog. Lipid Res..

[55] Coelho N.R., Pimpão A.B., Correia M.J., Rodrigues T.C., Monteiro E.C., Morello J., Pereira S.A. (2022). Pharmacological blockage of the AHR-CYP1A1 axis: A call for in vivo evidence. J. Mol. Med..

[56] Kim K., Kwon J.S., Ahn C., Jeung E.B. (2022). Endocrine-Disrupting Chemicals and Their Adverse Effects on the Endoplasmic Reticulum. Int. J. Mol. Sci..

[57] Kazzaz S.A., Tawil J., Harhaj E.W. (2024). The aryl hydrocarbon receptor-interacting protein in cancer and immunity: Beyond a chaperone protein for the dioxin receptor. J. Biol. Chem..

[58] Pappas B., Yang Y., Wang Y., Kim K., Chung H.J., Cheung M., Ngo K., Shinn A., Chan W.K. (2018). p23 protects the human aryl hydrocarbon receptor from degradation via a heat shock protein 90-independent mechanism. Biochem. Pharmacol..

[59] Wang Y., Qian H. (2021). Phthalates and Their Impacts on Human Health. Healthcare.

[60] Baker B.H., Melough M.M., Paquette A.G., Barrett E.S., Day D.B., Kannan K., Nguyen R.H., Bush N.R., LeWinn K.Z., Carroll K.N. (2024). Ultra-processed and fast food consumption, exposure to phthalates during pregnancy, and socioeconomic disparities in phthalate exposures. Environ. Int..

[61] Tagne-Fotso R., Riou M., Saoudi A., Zeghnoun A., Frederiksen H., Berman T., Montazeri P., Andersson A.-M., Rodriguez-Martin L., Akesson A. (2024). Exposure to bisphenol A in European women from 2007 to 2014 using human biomonitoring data-The European Joint Programme HBM4EU. Environ. Int..

[62] Dalamaga M., Kounatidis D., Tsilingiris D., Vallianou N.G., Karampela I., Psallida S., Papavassiliou A.G. (2024). The role of endocrine disruptors bisphenols and phthalates in obesity: Current evidence, perspectives and controversies. Int. J. Mol. Sci..

[63] González-Casanova J.E., Bermúdez V., Caro Fuentes N.J., Angarita L.C., Caicedo N.H., Rivas Muñoz J., Rojas-Gómez D.M. (2023). New evidence on BPA’s role in adipose tissue development of proinflammatory processes and its relationship with obesity. Int. J. Mol. Sci..

[64] Dong Z., He L., Wu J., Xie C., Geng S., Wu J., Zhong C., Li X. (2025). Bisphenol A-Induced Cancer-Associated Adipocytes Promotes Breast Carcinogenesis via CXCL12/AKT Signaling. Mol. Cell. Endocrinol..

[65] Bokobza E., Hinault C., Tiroille V., Clavel S., Bost F., Chevalier N. (2021). The adipose tissue at the crosstalk between EDCs and cancer development. Front. Endocrinol..

[66] Banerjee O., Paul T., Singh S., Maji B.K., Mukherjee S. (2025). Individual and combined antagonism of aryl hydrocarbon receptor (AhR) and estrogen receptors (ERs) offers distinct level of protection against Bisphenol A (BPA)-induced pancreatic islet cell toxicity in mice. Naunyn Schmiedebergs Arch. Pharmacol..

[67] Szaefer H., Licznerska B., Baer-Dubowska W. (2024). The Aryl Hydrocarbon Receptor and Its Crosstalk: A Chemopreventive Target of Naturally Occurring and Modified Phytochemicals. Molecules.

[68] Donini C.F., El Helou M., Wierinckx A., Győrffy B., Aires S., Escande A., Croze S., Clezardin P., Lachuer J., Diab-Assaf M. (2020). Long-Term Exposure of Early-Transformed Human Mammary Cells to Low Doses of Benzo[a]pyrene and/or Bisphenol A Enhances Their Cancerous Phenotype via an AhR/GPR30 Interplay. Front. Oncol..

[69] Kodila A., Franko N., Sollner Dolenc M. (2023). A review on immunomodulatory effects of BPA analogues. Arch. Toxicol..

[70] Primost M.A., Chierichetti M.A., Castaños C., Bigatti G., Miglioranza K.S.B. (2024). Persistent Organic Pollutants (POPs), Current Use Pesticides (CUPs) and Polycyclic Aromatic Hydrocarbons (PAHs) in edible marine invertebrates from a Patagonian harbor. Mar. Pollut. Bull..

[71] Ditchfield C., Kushida M.M., Mazalli M.R., Sobral P.J.A. (2023). Can Chocolate Be Classified as an Ultra-Processed Food? A Short Review on Processing and Health Aspects to Help Answer This Question. Foods.

[72] Muncke J., Touvier M., Trasande L., Scheringer M. (2025). Health impacts of exposure to synthetic chemicals in food. Nat. Med..

[73] Tian Z., Chen S., Shi Y., Wang P., Wu Y., Li G. (2023). Dietary advanced glycation end products (dAGEs): An insight between modern diet and health. Food Chem..

[74] Yuan X., Nie C., Liu H., Ma Q., Peng B., Zhang M., Chen Z., Li J. (2023). Comparison of metabolic fate, target organs, and microbiota interactions of free and bound dietary advanced glycation end products. Crit. Rev. Food Sci. Nutr..

[75] Zinöcker M.K., Lindseth I.A. (2018). The Western diet–microbiome–host interaction and its role in metabolic disease. Nutrients.

[76] Parada Venegas D., De la Fuente M.K., Landskron G., González M.J., Quera R., Dijkstra G., Harmsen H.J.M., Faber K.N., Hermoso M.A. (2019). Short chain fatty acids (SCFAs)-mediated gut epithelial and immune regulation and its relevance for inflammatory bowel diseases. Front. Immunol..

[77] Zmora N., Suez J., Elinav E. (2019). You are what you eat: Diet, health and the gut microbiota. Nat. Rev. Gastroenterol. Hepatol..

[78] Chassaing B., Koren O., Goodrich J.K., Poole A.C., Srinivasan S., Ley R.E., Gewirtz A.T. (2015). Dietary emulsifiers impact the mouse gut microbiota promoting colitis and metabolic syndrome. Nature.

[79] Wirbel J., Pyl P.T., Kartal E., Zych K., Kashani A., Milanese A., Fleck J.S., Voigt A.Y., Palleja A., Ponnudurai R. (2019). Meta-analysis of fecal metagenomes reveals global microbial signatures that are specific for colorectal cancer. Nat. Med..

[80] Aguilera M., Gálvez-Ontiveros Y., Rivas A. (2020). Endobolome, a new concept for determining the influence of microbiota disrupting chemicals (MDC) in relation to specific endocrine pathogenesis. Front. Microbiol..

[81] Gopalakrishnan V., Helmink B.A., Spencer C.N., Reuben A., Wargo J.A. (2018). The influence of the gut microbiome on cancer, immunity, and cancer immunotherapy. Cancer Cell.

[82] Huang G., Zheng Y., Zhang N., Huang G., Zhang W., Li Q., Ren X. (2024). Desulfovibrio vulgaris caused gut inflammation and aggravated DSS-induced colitis in C57BL/6 mice model. Gut Pathog..

[83] Liu Y., Zhang S., Zhou W., Hu D., Xu H., Ji G. (2022). Secondary bile acids and tumorigenesis in colorectal cancer. Front. Oncol..

[84] Yang S., Dai H., Lu Y., Li R., Gao C., Pan S. (2022). Trimethylamine N-oxide promotes cell proliferation and angiogenesis in colorectal cancer. J. Immunol. Res..

[85] Gubatan J., Boye T.L., Temby M., Sojwal R.S., Holman D.R., Sinha S.R., Rogalla S.R., Nielsen O.H. (2022). Gut microbiome in inflammatory bowel disease: Role in pathogenesis, dietary modulation, and colitis-associated colon cancer. Microorganisms.

[86] O’Toole P.W., Marchesi J.R., Hill C. (2017). Next-generation probiotics: The spectrum from probiotics to live biotherapeutics. Nat. Microbiol..

[87] Bevilacqua A., Speranza B., Racioppo A., Santillo A., Albenzio M., Derossi A., Caporizzi R., Francavilla M., Racca D., Flagella Z. (2024). Ultra-Processed Food and Gut Microbiota: Do Additives Affect Eubiosis? A Narrative Review. Nutrients.

[88] Alcaire F., Giménez A., Ares G. (2024). Food Additives Associated with Gut Dysbiosis in Processed and Ultra-Processed Products Commercialized in the Uruguayan Market. Food Res. Int..

[89] Gonza I., Goya-Jorge E., Douny C., Boutaleb S., Taminiau B., Daube G., Scippo M., Louis E., Delcenserie V. (2024). Food Additives Impair Gut Microbiota from Healthy Individuals and IBD Patients in a Colonic In Vitro Fermentation Model. Food Res. Int..

[90] Li P., Qu R., Li M., Sheng P., Jin L., Huang X., Xu Z.Z. (2024). Impacts of Food Additives on Gut Microbiota and Host Health. Food Res. Int..

[91] Chassaing B., Compher C., Bonhomme B., Liu Q., Tian Y., Walters W., Nessel L., Delaroque C., Hao F., Gershuni V. (2022). Randomized Controlled-Feeding Study of Dietary Emulsifier Carboxymethylcellulose Reveals Detrimental Impacts on the Gut Microbiota and Metabolome. Gastroenterology.

[92] Rosés C., Nieto J.A., Viadel B., Gallego E., Romo-Hualde A., Streitenberger S., Milagro F.I., Barceló A. (2021). An In Vitro Protocol to Study the Modulatory Effects of a Food or Biocompound on Human Gut Microbiome and Metabolome. Foods.

[93] Lerma-Aguilera A.M., Pérez-Burillo S., Navajas-Porras B., León E.D., Ruíz-Pérez S., Pastoriza S., Jiménez-Hernández N., Cämmerer B.-M., Rufián-Henares J.Á., Gosalbes M.J. (2024). Effects of Different Foods and Cooking Methods on the Gut Microbiota: An In Vitro Approach. Front. Microbiol..

[94] Karl J.P., Armstrong N.J., Player R.A., Rood J.C., Soares J.W., McClung H.L. (2022). The Fecal Metabolome Links Diet Composition, Foacidic Positive Ion Conditions, Chromatographicallyod Processing, and the Gut Microbiota to Gastrointestinal Health in a Randomized Trial of Adults Consuming a Processed Diet. J. Nutr..

[95] Leeming E.R., Johnson A.J., Spector T.D., Le Roy C.I. (2019). Effect of Diet on the Gut Microbiota: Rethinking Intervention Duration. Nutrients.

[96] Gerasimidis K., Bryden K., Chen X., Papachristou E., Verney A., Roig M., Hansen R., Nichols B., Papadopoulou R., Parrett A. (2020). The Impact of Food Additives, Artificial Sweeteners and Domestic Hygiene Products on the Human Gut Microbiome and Its Fibre Fermentation Capacity. Eur. J. Nutr..

[97] Hussain S., Tulsyan S., Dar S.A., Sisodiya S., Abiha U., Kumar R., Mishra B.N., Haque S. (2022). Role of epigenetics in carcinogenesis: Recent advancements in anticancer therapy. Semin. Cancer Biol..

[98] Barrero M.J., Cejas P., Long H.W., Ramirez de Molina A. (2022). Nutritional epigenetics in cancer. Adv. Nutr..

[99] Whelan K., Bancil A.S., Lindsay J.O., Chassaing B. (2024). Ultra-processed foods and food additives in gut health and disease. Nat. Rev. Gastroenterol. Hepatol..

[100] Isaksen I.M., Dankel S.N. (2023). Ultra-processed food consumption and cancer risk: A systematic review and meta-analysis. Clin. Nutr..

[101] Srour B., Kordahi M.C., Bonazzi E., Deschasaux-Tanguy M., Touvier M., Chassaing B. (2022). Ultra-processed foods and human health: From epidemiological evidence to mechanistic insights. Lancet Gastroenterol. Hepatol..

[102] Pértille F., Da Silva V.H., Johansson A.M., Lindström T., Wright D., Coutinho L.L., Jensen P., Guerrero-Bosagna C. (2019). Mutation dynamics of CpG dinucleotides during a recent event of vertebrate diversification. Epigenetics.

[103] Bishop K.S., Ferguson L.R. (2015). The interaction between epigenetics, nutrition and the development of cancer. Nutrients.

[104] Zhong F., Lin Y., Zhao L., Yang C., Ye Y., Shen Z. (2023). Reshaping the tumour immune microenvironment in solid tumours via tumour cell and immune cell DNA methylation: From mechanisms to therapeutics. Br. J. Cancer.

[105] Bhootra S., Jill N., Shanmugam G., Rakshit S., Sarkar K. (2023). DNA methylation and cancer: Transcriptional regulation, prognostic, and therapeutic perspective. Med. Oncol..

[106] Li N., Song K., Chen H., Dai M. (2025). Advance and challenge of DNA methylation as cancer biomarkers for risk stratification, screening and early detection. J. Natl. Cancer Cent..

[107] Zaib S., Rana N., Khan I. (2022). Histone modifications and their role in epigenetics of cancer. Curr. Med. Chem..

[108] Neganova M.E., Klochkov S.G., Aleksandrova Y.R., Aliev G. (2022). Histone modifications in epigenetic regulation of cancer: Perspectives and achieved progress. Semin. Cancer Biol..

[109] Zhang Y., Sun Z., Jia J., Du T., Zhang N., Tang Y., Fang Y., Fang D. (2021). Overview of histone modification. Adv. Exp. Med. Biol..

[110] Zhang Z., Zhang J., Diao L., Han L. (2021). Small non-coding RNAs in human cancer: Function, clinical utility, and characterisation. Oncogene.

[111] Zhang X., Xu X., Song J., Xu Y., Qian H., Jin J., Liang Z.F. (2023). Non-coding RNAs’ function in cancer development, diagnosis and therapy. Biomed. Pharmacother..

[112] Romano G., Veneziano D., Acunzo M., Croce C.M. (2017). Small non-coding RNA and cancer. Carcinogenesis.

[113] Ferrante M., Cristaldi A., Oliveri Conti G. (2021). Oncogenic role of miRNA in environmental exposure to plasticisers: A systematic review. J. Pers. Med..

[114] Llauradó-Pont J., Stratakis N., Fiorito G., Handakas E., Neumann A., Barros H., Brantsæter A.L., Chang K., Chatzi L., Felix J.F. (2025). A Meta-Analysis of Epigenome-Wide Association Studies of Ultra-Processed Food Consumption with DNA Methylation in European Children. Clin. Epigenetics.

[115] Edalati S., Bagherzadeh F., Asghari Jafarabadi M., Ebrahimi-Mamaghani M. (2021). Higher Ultra-Processed Food Intake Is Associated with Higher DNA Damage in Healthy Adolescents. Br. J. Nutr..

[116] Freitas R.D.S., da Silva J. (2025). Impact of Ultra-Processed Foods on Human Health: A Comprehensive Review of Genomic Instability and Molecular Mechanisms. Nutrition.

[117] Gerhauser C. (2018). Impact of Dietary Gut Microbial Metabolites on the Epigenome. Philos. Trans. R. Soc. Lond. B Biol. Sci..

[118] Zhang Y., Giovannucci E.L. (2023). Ultra-Processed Foods and Health: A Comprehensive Review. Crit. Rev. Food Sci. Nutr..

[119] Milagro F.I., Mansego M.L., De Miguel C., Martínez J.A. (2013). Dietary Factors, Epigenetic Modifications and Obesity Outcomes: Progresses and Perspectives. Mol. Asp. Med..

[120] Liu W., Deng Y., Li Z., Chen Y., Zhu X., Tan X., Cao G. (2021). Cancer Evo-Dev: A theory of inflammation-induced oncogenesis. Front. Immunol..

[121] Mella C., Tsarouhas P., Brockwell M., Ball H.C. (2025). The role of chronic inflammation in pediatric cancer. Cancers.

[122] Mohammed D.M., Abdelgawad M.A., Ghoneim M.M., Alhossan A., Al-Serwi R.H., Farouk A. (2024). Impact of some natural and artificial sweeteners consumption on different hormonal levels and inflammatory cytokines in male rats: In vivo and in silico studies. ACS Omega.

[123] Yang L., Wang S., Jin J., Wang J., Chen W., Xue Y., Sheng L., Zhai Y., Yao W. (2024). Sucralose triggers insulin resistance leading to follicular dysplasia in mice. Reprod. Toxicol..

[124] Tristan Asensi M., Napoletano A., Sofi F., Dinu M. (2023). Low-grade inflammation and ultra-processed foods consumption: A review. Nutrients.

[125] Sylvetsky A.C., Wang Y., Reddy A.G., Um C.Y., Hodge R.A., Lichtman C., Mitchell D., Nanavati A., Pollak M., Wang Y. (2025). Nonnutritive sweetener consumption, metabolic risk factors, and inflammatory biomarkers among adults in the Cancer Prevention Study-3 Diet Assessment Sub-Study. J. Nutr..

[126] Sánchez-Tapia M., Miller A.W., Granados-Portillo O., Tovar A.R., Torres N. (2020). The development of metabolic endotoxemia is dependent on the type of sweetener and the presence of saturated fat in the diet. Gut Microbes.

[127] Bujtor M. (2021). Can dietary intake protect against low-grade inflammation in children and adolescents?. Brain Behav. Immun. Health.

[128] Chavez-Dominguez R., Perez-Medina M., Aguilar-Cazares D., Galicia-Velasco M., Meneses-Flores M., Islas-Vazquez L., Camarena A., Lopez-Gonzalez J.S. (2021). Old and new players of inflammation and their relationship with cancer development. Front. Oncol..

[129] Harris H.R., Willett W.C., Vaidya R.L., Michels K.B. (2017). An adolescent and early adulthood dietary pattern associated with inflammation and the incidence of breast cancer. Cancer Res..

[130] Suzuki S., Katagiri R., Yamaji T., Sawada N., Imatoh T., Ihira H., Inoue M., Tsugane S., Iwasaki M., Japan Public Health Center-Based Prospective Study Group (2022). Association between C-reactive protein and risk of overall and 18 site-specific cancers in a Japanese case-cohort. Br. J. Cancer.

[131] Yang K., Song X., Cheng C., Shi Q., Li X., Long J., Yang H., Chen S. (2025). Association between dietary inflammatory potential and liver cancer risk: A systematic review and dose-response meta-analysis. Nutr. Cancer.

[132] Ramal-Sanchez M., Bravo-Trippetta C., D’aNtonio V., Corvaglia E., Kämpfer A.A.M., Schins R.P.F., Serafini M., Angelino D. (2025). Development and Assessment of an Intestinal Tri-Cellular Model to Investigate the Pro/Anti-Inflammatory Potential of Digested Foods. Front. Immunol..

[133] Vahid F., Krischler P., Leners B., Bohn T. (2024). Effect of Digested Selected Food Items on Markers of Oxidative Stress and Inflammation in a Caco-2-Based Human Gut Epithelial Model. Antioxidants.

[134] Le N.P.K., Altenburger M.J., Lamy E. (2023). Development of an Inflammation-Triggered In Vitro “Leaky Gut” Model Using Caco-2/HT29-MTX-E12 Combined with Macrophage-like THP-1 Cells or Primary Human-Derived Macrophages. Int. J. Mol. Sci..

[135] Nogueira S., Barbosa J., Faria J., Sá S.I., Cardoso A., Soares R., Fonseca B.M., Leal S. (2022). Unhealthy Diets Induce Distinct and Regional Effects on Intestinal Inflammatory Signalling Pathways and Long-Lasting Metabolic Dysfunction in Rats. Int. J. Mol. Sci..

[136] Viennois E., Bretin A., Dubé P.E., Maue A.C., Dauriat C.J.G., Barnich N., Gewirtz A.T., Chassaing B. (2020). Dietary Emulsifiers Directly Impact Adherent-Invasive *E. coli* Gene Expression to Drive Chronic Intestinal Inflammation. Cell Rep..

[137] Mignogna C., Costanzo S., Di Castelnuovo A., Ruggiero E., Shivappa N., Hebert J.R., Esposito S., De Curtis A., Persichillo M., Cerletti C. (2022). The Inflammatory Potential of the Diet as a Link between Food Processing and Low-Grade Inflammation: An Analysis on 21,315 Participants to the Moli-sani Study. Clin. Nutr..

[138] Vitale M., Costabile G., Testa R., D’Abbronzo G., Nettore I.C., Macchia P.E., Giacco R. (2024). Ultra-processed foods and human health: A systematic review and meta-analysis of prospective cohort studies. Adv. Nutr..

[139] Forde C.G. (2023). Beyond ultra-processed: Considering the future role of food processing in human health. Proc. Nutr. Soc..

[140] Anastasiou I.A., Kounatidis D., Vallianou N.G., Skourtis A., Dimitriou K., Tzivaki I., Tsioulos G., Rigatou A., Karampela I., Dalamaga M. (2025). Beneath the surface: The emerging role of ultra-processed foods in obesity-related cancer. Curr. Oncol. Rep..

[141] Morys F., Kanyamibwa A., Fängström D., Tweedale M., Pastor-Bernier A., Azizi H., Liu L., Horstmann A., Dagher A. (2025). Ultra-processed food consumption affects structural integrity of feeding-related brain regions independent of and via adiposity. NPJ Metab. Health Dis..

[142] Martínez Leo E.E., Peñafiel A.M., Hernández Escalante V.M., Cabrera Araujo Z.M. (2021). Ultra-processed diet, systemic oxidative stress, and breach of immunologic tolerance. Nutrition.

[143] Lustig R.H. (2020). Ultraprocessed food: Addictive, toxic, and ready for regulation. Nutrients.

[144] Baghy K., Ladányi A., Reszegi A., Kovalszky I. (2023). Insights into the tumor microenvironment—Components, functions and therapeutics. Int. J. Mol. Sci..

[145] Chen X., Zhang Z., Yang H., Qiu P., Wang H., Wang F., Zhao Q., Fang J., Nie J. (2020). Consumption of ultra-processed foods and health outcomes: A systematic review of epidemiological studies. Nutr. J..

[146] Harris E. (2024). Ultraprocessed foods linked with 32 types of health problems. JAMA.

[147] De Paula L.C.P., Alves C. (2024). Food packaging and endocrine disruptors. J. Pediatr..

[148] Valicente V.M., Peng C.H., Pacheco K.N., Lin L., Kielb E.I., Dawoodani E., Abdollahi A., Mattes R.D. (2023). Ultraprocessed foods and obesity risk: A critical review of reported mechanisms. Adv. Nutr..

[149] Tobias D.K., Hall K.D. (2021). Eliminate or reformulate ultra-processed foods? Biological mechanisms matter. Cell Metab..

[150] Wang L., Du M., Wang K., Shen W., Lu D., Zheng Y., Song M., Cao Y., Smith-Warner S.A., Ogino S. (2022). Association of ultra-processed food consumption with colorectal cancer risk among men and women: Results from three prospective US cohort studies. BMJ.

[151] Jafari F., Yarmand S., Nouri M., Akbari M.E., Hadaegh F., Azizi F., Mirmiran P. (2023). Ultra-processed food intake and risk of colorectal cancer: A matched case-control study. Nutr. Cancer.

[152] Zhong G.C., Gu H.T., Peng Y., Wang K., Wu Y.Q., Hu T.Y., Jing F.C., Hao F.B. (2021). Association of ultra-processed food consumption with cardiovascular mortality in the US population: Long-term results from a large prospective multicenter study. Int. J. Behav. Nutr. Phys. Act..

